# Bayesian evidence for the neural dissociation between finger and hand imitation skills

**DOI:** 10.1162/imag_a_00342

**Published:** 2024-11-01

**Authors:** Hannah Rosenzopf, Lisa Röhrig, Georg Goldenberg, Hans-Otto Karnath

**Affiliations:** Centre of Neurology, Division of Neuropsychology, Hertie Institute for Clinical Brain Research, University of Tübingen, Tübingen, Germany; Neurological Department, Technical University Munich, Munich, Germany; Department of Psychology, University of South Carolina, Columbia, SC, United States

**Keywords:** apraxia, limb movement, meaningless gestures, praxis skills, voxel-based lesion symptom mapping, Bayesian hypothesis testing, motor system

## Abstract

For limb apraxia—a heterogeneous disorder of higher motor cognition following stroke—an enduring debate has arisen regarding the existence of dissociating neural correlates for finger and hand gestures in the left hemisphere. We re-assessed this question asking whether previous attempts analyzing pooled samples of patients with deficits in only one and patients with deficits in both imitation types might have led to systematically biased results. We conducted frequentist and Bayesian, voxelwise, and regionwise lesion symptom mappings on (i) the full sample (N = 96) in which all patients with hand and/or finger imitation apraxia as well as without apraxia were included and (ii) three sub-samples, which excluded those patients from the full sample showing isolated hand imitation deficits, isolated finger imitation deficits or shared (finger and hand) imitation deficits. Anatomical analyses revealed a cortical dissociation of finger imitation deficits (located more anteriorly) and of hand imitation deficits (located more posteriorly). The presence of patients with shared deficits did, indeed, dilute associations that appeared stronger in the respective isolated samples. Also, brain regions truly associated with hand imitation deficits showed a positive bias for finger imitation deficits, when the sample contained patients with shared deficits. In addition, our frequentist parameters uncovered that some of our Bayesian evidence supported reverse associations (damage protecting from rather than increasing the deficit). Anatomo-behavioral analyses that analyze patients with shared (hand and finger) and isolated (hand or finger) imitation deficits together in one sample do, indeed, lead to undesirable biases. This explains why some earlier studies failed to detect the apparent neural dissociation between hand and finger imitation deficits.

## 1 Introduction

Limb apraxia is a heterogeneous disorder of higher motor cognition, impairing the execution of different types of actions (Randerath, 2023). The earliest research distinguished between ideational, ideomotor, and limb kinetic apraxia (Liepman, 1920). In an attempt to create a more symptom-oriented classification,Goldenberg (2011)introduced a subdivision into the apraxias of (1) tool use, (2) gesture production, and (3) imitation. The apraxia of imitation is further subdivided based on the affected body part, that is, the hand (De Renzi et al., 1980;Goldenberg, 1996;Goldenberg & Karnath, 2006;Kimura & Archibald, 1974;Lehmkuhl et al., 1982), fingers (Goldenberg, 1996;Goldenberg & Karnath, 2006;Goldenberg & Strauss, 2002), legs (Lehmkuhl et al., 1982), feet (Goldenberg & Strauss, 2002), as well as different facial features (Bizzozero et al., 2000;Lehmkuhl et al., 1982).

While hemispheric differences based on the impaired body part support behavioral dissociations (Bizzozero et al., 2000;Goldenberg, 1996;Goldenberg & Strauss, 2002;Lausberg & Cruz, 2004), a lasting controversy about whether or not finger and hand gestures have dissociating neural correlates in the left hemisphere has emerged. Reports of dissociating neural correlates for finger and hand imitation deficits in the left hemisphere (Goldenberg, 1996;Goldenberg & Karnath, 2006;Haaland et al., 2000) seemingly contradict more recent accounts, which failed to uncover such dissociation (Achilles et al., 2017;Hoeren et al., 2014). In particular,Achilles et al. (2017)aimed at eliminating this ambiguity once and for all by applying Bayesian statistics, which they judge as particular beneficial in this context, due to its ability to uncover evidence for both alternative and null hypothesis. Their results, derived from a large sample of 257 left hemisphere stroke patients, discount previous evidence for the dissociation, arguing that it had been reported only by small samples that had not performed any statistical evaluations. However, another crucial difference between the latter studies demonstrating dissociating neural substrates and those that did not, including the one byAchilles et al. (2017), is a fundamentally different sampling approach. While the earlier descriptive studies (that found a dissociation) exclusively compared patients with isolated hand and finger imitation deficits to controls without the respective deficit, the more recent statistical analyses (that did not find a dissociation) assigned patients with both shared and isolated deficits to the same group.

Grouping patients with shared and isolated imitation deficits together could potentially be problematic, in particular when investigating the existence of a task dissociation. We know that the lesions of patients with an isolated deficit can, in theory, only be associated with that deficit but not the other (which in itself should already be considered as support for a dissociation). Lesions of patients with a shared deficit of hand and finger imitation skills could contain what henceforth will be considered*shared areas*(i.e., areas whose damage leads to a simultaneous deficit in hand and finger imitation skills), or will at least contain voxels of both kinds of*isolated areas*(i.e., areas leading to only hand or only finger imitation deficits). In scenario one, a voxel truly causing an isolated deficit will reduce the power of voxels truly associated with a shared deficit and vice versa. In scenario two, patients with a shared deficit will have simultaneous damage in*isolated hand*- and*isolated finger areas*. That is, voxels leading to the finger deficit will unavoidably be represented in patients’ lesion-associations with the hand deficit and vice versa, therefore potentially introducing a systematic bias. Both scenarios could lead to the wrongful rejection of a truly existing dissociation, the former by uncovering only*shared areas*but not the underpowered isolated ones, the latter by erroneously associating*isolated areas*for one deficit also with the other, due to their frequent co-occurrence in patients with a shared deficit.

Achilles et al. (2017)assigned damage to brain regions associated with imitation skills a higher susceptibility to hand gesture deficits and argued that finger deficits might accompany particularly pronounced problems with hand imitation. At the same time, the authors reported 10 patients with the opposite dissociation (i.e., patients with an isolated finger imitation deficit). They argued that eight of those cases, who had a very mild isolated finger imitation deficit, might be considered noise by the model but admit that their rationale does not sufficiently explain the remaining two cases with a severe isolated finger imitation deficit. In light of the methodological dependencies described above, a potential alternative explanation could be that the results obtained byAchilles et al. (2017)reflect the larger subgroups with shared deficits, which might, indeed, have a higher susceptibility for hand than for finger gestures. The higher frequency of*shared areas*could have outpowered*isolated areas*. Alternatively, isolated hand areas could have been wrongly associated to finger imitation deficits.

In the present study, we investigated whether appropriate subsampling can, indeed, uncover dissociating neural correlates of hand and finger imitation deficits that are masked in a joint analysis of patients with dissociating and shared imitation deficits. We first replicated the hand and finger imitation analyses reported byAchilles et al. (2017)in which all patients with hand and/or finger imitation apraxia as well as patients without apraxia were included (‘*full sample*’). We then performed adaptations of these analyses with different sub-samples. In two of these sub-samples, we excluded patients with isolated imitation deficits from the full sample. For a third sub-sample, we excluded all patients from the full sample with a shared deficit, that is, patients who presented with a hand imitation deficit combined with finger imitation deficit. Beyond, we applied both frequentist and Bayesian approaches on both the voxel and the region level. We hypothesized that the sub-samples will uncover dissociating voxels and brain regions with higher sensitivity than in the (negatively biased) full sample. Second, in the regionwise analyses (which illustrate the effects of different sampling approaches), we expected analyses on finger imitation deficits on the full sample to show a positive bias for brain regions truly associated with hand imitation deficits and vice versa. Thirdly, (shared) areas leading to both hand as well as finger imitation deficits should have the best chance of being uncovered in the sub-samples that exclude patients with isolated imitation deficits rather than in the full sample or the sub-sample that excludes patients with a shared (hand and finger) imitation deficit.

## 2 Methods

### 2.1 Participants

Data of 96 brain-damaged patients investigated at the Neuropsychological Department of the Bogenhausen Hospital in Munich, Germany, were analyzed retrospectively. The identical sample has previously been used to investigate associations between apraxia and aphasia (Goldenberg & Randerath, 2015). It includes 23 female and 73 male patients who had suffered a first-time left hemispheric stroke on average 13.3 weeks (range 3–58) before the behavioral examination. Seventy-three patients had suffered an ischemic stroke, 23 a hemorrhagic infarct. Additional demographic and clinical data, divided by patient sub-groups (see below), can be derived fromTable 1. Consent for the scientific reuse of their data was provided by the patients; We followed ethical standards as predefined by the revised Declaration of Helsinki.

**Table 1.** Descriptive statistics concerning relevant demographical and clinical data divided by patient sub-group.

	No deficit	Shared (hand & finger) deficit	Isolated deficit	
			Hand	Finger
N	40	23	25	8
Age (years)	55.9 (11.6)	61.6 (9.8)	54.0 (13.1)	57.5 (8.9)
Lesion size (mm³)	80.8 (47.3)	115.5 (93.1)	114.2 (87.3)	114.3 (79.1)
Time since lesion (weeks)	12.8 (19)	7 (4.1)	18.1 (18.8)	19.3 (17.2)
Imitation hand score (max. 20)	19 (0.8)	12.3 (4.6)	15.9 (3.2)	19.1 (0.6)
Imitation finger score (max. 20)	18.9 (1.1)	12 (3.7)	18.6 (1.0)	13.5 (2.7)
Pantomime score (max. 55)	45.7 (8.0)	33.3 (13.9)	44.8 (11.1)	42 (13.0)
Pantomime deficit (score <45) %	30	78.3	44.8	37.5
Hemiparesis %	50	47	48	75
Aphasia * %	95	87	96	100

No deficit, patients with scores above the cut-off in both imitation tests; Shared deficit, patients with scores below the cut-off in both imitation tests; Isolated deficit, patients with scores below the cut-off in only one imitation test but not the other. Unless otherwise stated, values refer to means (standard deviations).

* Aphasia diagnoses included a wide variety of aphasias such as Global, Broca’s, Wernicke’s, and Amnestic; their frequencies were similar across all four patient groups.

### 2.2 Behavioural testing and sub sampling

Diagnostic scores concerning the apraxia of the imitation of hand and finger gestures (Goldenberg, 1996) served as the two dependent variables in our analyses. For both tests, 10 hand or finger postures are modeled by the investigator. After having demonstrated a gesture, the investigator returns to a neutral posture before asking the patient to imitate the previously seen gesture. Two points are scored for a correct imitation at the first attempt. If the patient successfully imitated the gesture at the second attempt after seeing the gesture a second time, they received one point. This results in a maximum of 20 points for each test. The cut-off for pathological performance in the hand imitation test is <18, and the cut-off for pathological performance for the finger imitation test is <17 (Goldenberg, 1996). Other noteworthy assessments included the Aachen Aphasia Test (ATT) and a 20-item test assessing the apraxia of pantomime (Goldenberg, 2003;Goldenberg et al., 2007). The ‘*full sample*’ (N = 96) included all patients with hand and/or finger imitation apraxia as well as patients without apraxia. Based on this ‘*full sample*’, three different sub-samples were formed. The ‘*shared hand sub-sample*’ (N = 71) did not contain those patients from the ‘*full sample*’ with an isolated hand imitation deficit. The ‘*shared finger sub-sample*’ (N = 88) did not contain those patients from the ‘*full sample*’ with an isolated finger imitation deficit. The ‘*isolated sub-sample*’ (N = 73) did not contain those patients from the ‘*full sample*’ with a shared deficit, that is, patients who had both finger and a hand imitation deficit simultaneously.

### 2.3 Imaging and Image processing

CT (N = 12) and MRI (N = 84) imaging obtained within 3 weeks from the behavioural testing was used to manually map patients’ lesions on axial slices of a T1-weighted template in MNI space, which is accessible via MRIcro software (Rorden & Brett, 2000;http://people.cas.sc.edu/rorden/mricro/index.html). Eleven slices incrementing in steps of 8 mm from z-coordinate -40 to 40 plus a twelfth one representing z-coordinate 50 were used to map patients’ lesions to the respective (or the closest) available axial slice. These slices were 3-dimensionally reconstructed using a MATLAB script previously used for this purpose (Klingbeil et al., 2020). This script expands the existing slices along the z-axis and smooths the resulting images with a Gaussian kernel. The lesions were then (re)binarized at a threshold of 0.45 (for details seeKlingbeil et al., 2020). Lesion overlays divided by the four patient sub-groups (see above) are illustrated inFigure 1.

**Fig. 1.**
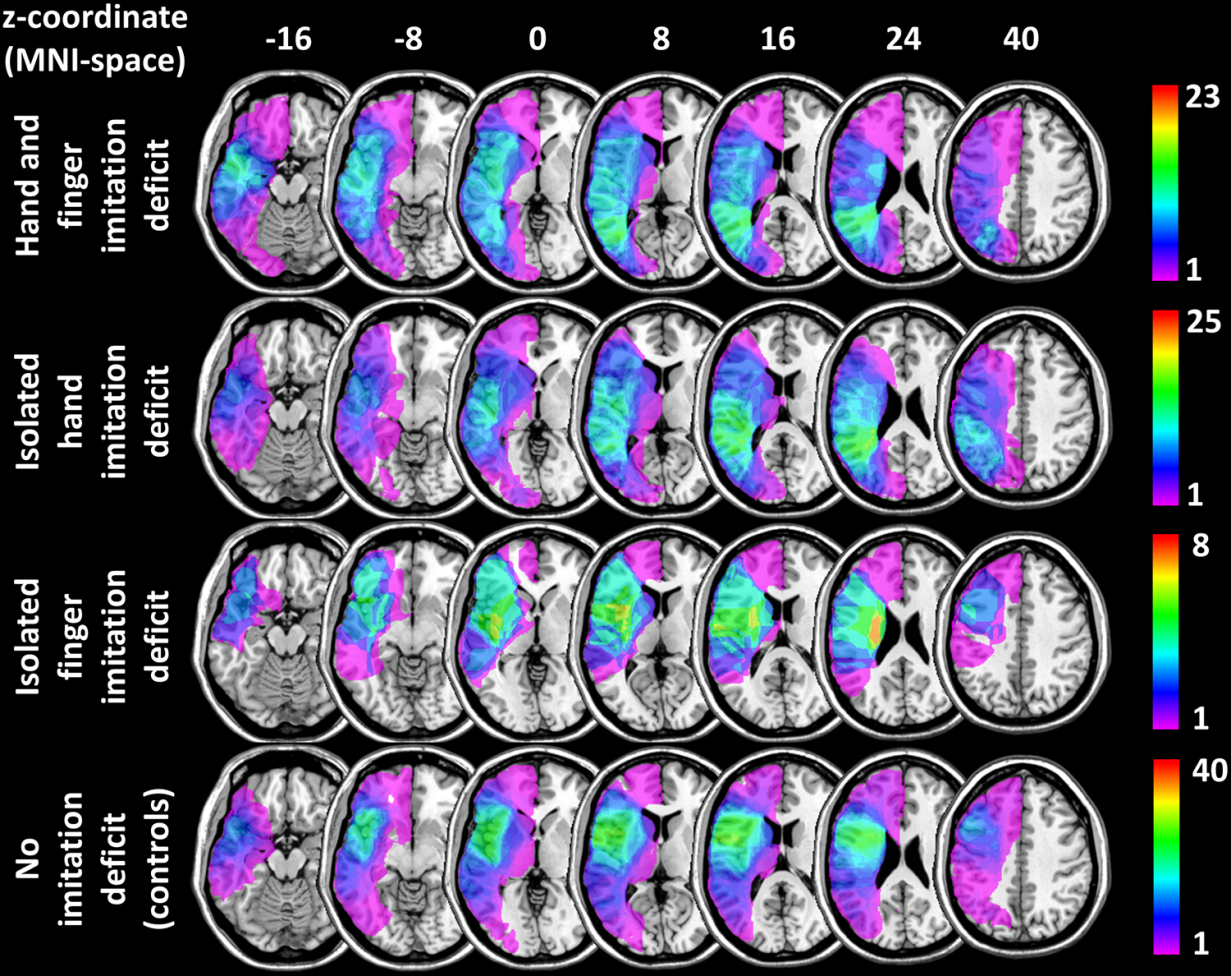
Lesion overlays for different patient groups.

### 2.4 Data analysis

Depending on the imitation type investigated, voxelwise as well as regionwise brain-behavior associations were modeled using either the hand or the finger imitation score as the dependent variable. The ‘*full sample*’ and the ‘*isolated sample*’ were used for the analyses of both hand and finger scores. The ‘*shared hand sub-sample*’ was only used to investigate the hand score. The ‘*shared finger sub-sample*’ was only used to examine the finger score. Voxelwise lesion-symptom mapping (VLSM) was applied based on frequentist mass-univariate General Linear Models (GLMs) using NiiStat (https://github.com/neurolabusc/NiiStat) and Bayesian GLMs using the Bayesian lesion-deficit inference (BLDI) software (Sperber et al., 2023). Both approaches were set to include voxels damaged in at least 5 patients. Results of the frequentist approach were corrected for multiple comparisons using False Discovery Rate at a corrected p < .01 (FDR;Benjamini & Hochberg, 1995). The statistical parameters returned by NiiStat are z-scores. To keep results comparable to those in the study byAchilles et al. (2017), we adopted their definition of strong evidence as a Bayes Factor (BF) >20. Unlike the BLDI, which uses the 10th logarithm,Achilles et al. (2017)used the natural logarithm, so we adapted the respective line in the BLDI script. In the applied version of the logBF, zero marks the border between evidence for H1 (represented by positive numbers) and evidence for H0 (represented by negative numbers), the equivalent to our cut-off (BF > 20) is a logBF > 2.99. VLSMs were conducted both with and without controlling for lesion size. Since patients with a shared deficit were on average tested earlier after stroke than the other patient groups, results for the samples containing them (i.e., the*full sample*,*shared hand sub-sample*and*shared finger sub-sample*) were repeated also controlled for time post stroke. Patient’s regionwise lesion load was assessed using custom scripts created using MATLAB 2019b, which overlapped each patient’s lesion with all left hemispheric parcels derived from the Desikan-Killiany atlas (Desikan et al., 2006) provided by the IIT Human Brain Atlas (v.5.0) (https://www.nitrc.org/projects/iit/). Anatomical terms throughout the manuscript follow the atlas and its labels. For the frequentist GLMs, we adapted customized MATLAB scripts, previously used for similar purposes (Röhrig et al., 2023). Correction for multiple comparisons was based on maximum-statistics permutation. 50,000 pseudo-random permutations were performed, and results were again reported for a corrected p < .01. In contrast to NiiStat, our MATLAB scripts return t-scores instead of z-scores. While the directionality here is the opposite (i.e., positive t-scores indicate the expected association between damage and worse performance), both are frequentist estimates of the associations and their directions. Since no script for a regionwise Bayesian GLM existed within the BLDI software, we customized the BLDI disconnection script to serve this purpose. Note that results in the main text are reported in BFs to avoid the underrepresentation of higher evidence, while logBFs were used for figures depicting VLSM results, to enhance the differentiability of the color gradients.

## 3 Results

### 3.1 Voxelwise lesion-symptom mapping

In order to investigate whether the ‘*full sample*’ indeed fails to uncover neural correlates, which emerge in the less biased sub samples and whether shared neural correlates emerge in the analyses on the ‘*shared hand sub-sample*’ and the ‘*shared finger sub-sample*’ but not the other analyses, we modeled voxelwise brain damage using the hand imitation score as the dependent variable in the ‘*full sample*’, the ‘*shared hand sub-sample*’, and the ‘*isolated sample*’ and the finger imitation score as the dependent variable in the ‘*full sample*’, the ‘*shared finger sub-sample*’, and ‘*isolated sample*’.

#### 3.1.1 Imitation of hand gestures

**Fig. 2.**
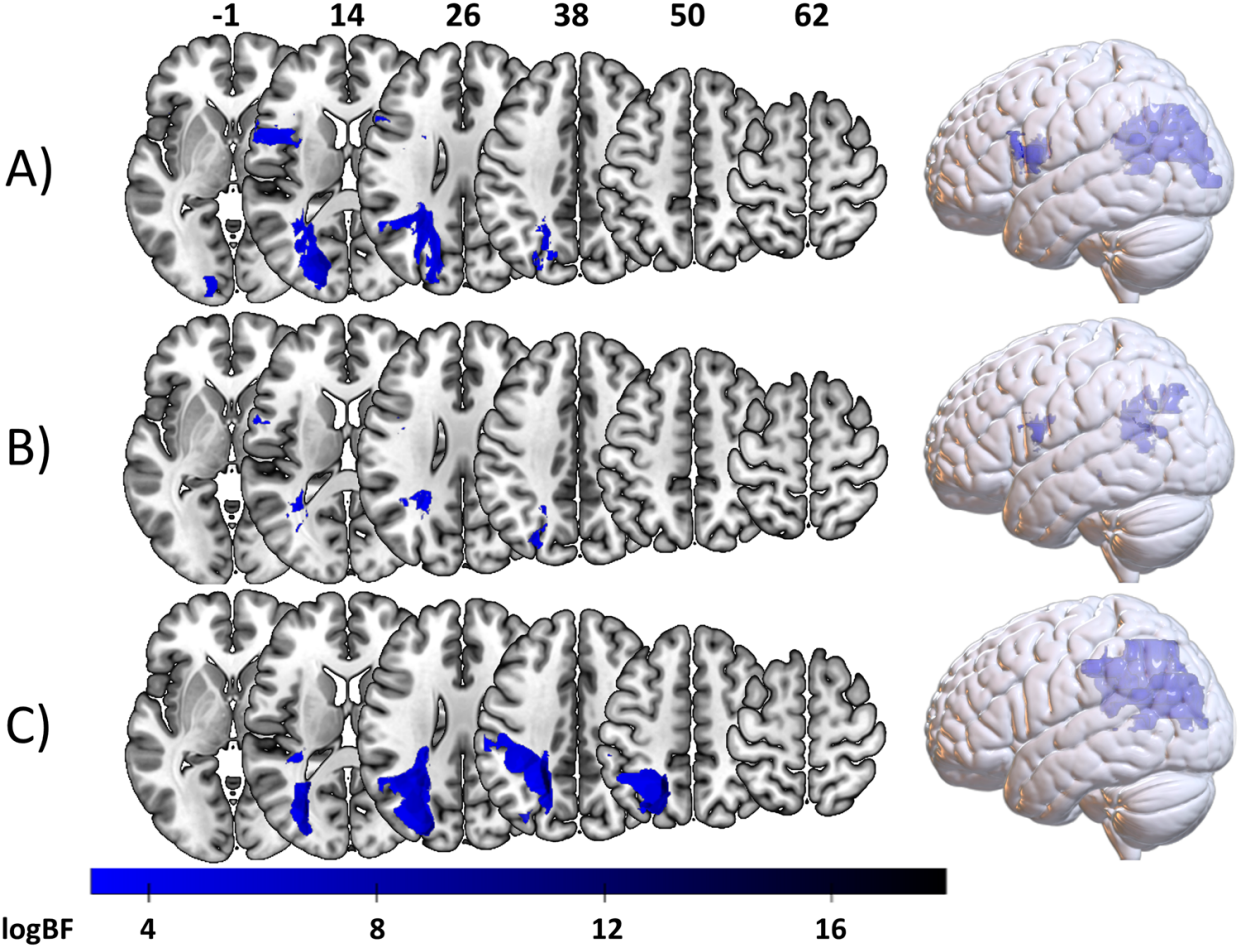
Bayesian associations between voxelwise brain damage and hand imitation deficits. Depicted are all voxels with a logBF > 2.99. Voxels associated with hand imitation deficits based on the VLSM on (A) the ‘*full sample*’, (B) the ‘*shared hand sub-sample*’, and (C) the ‘*isolated sub-sample*’.

**Fig. 3.**
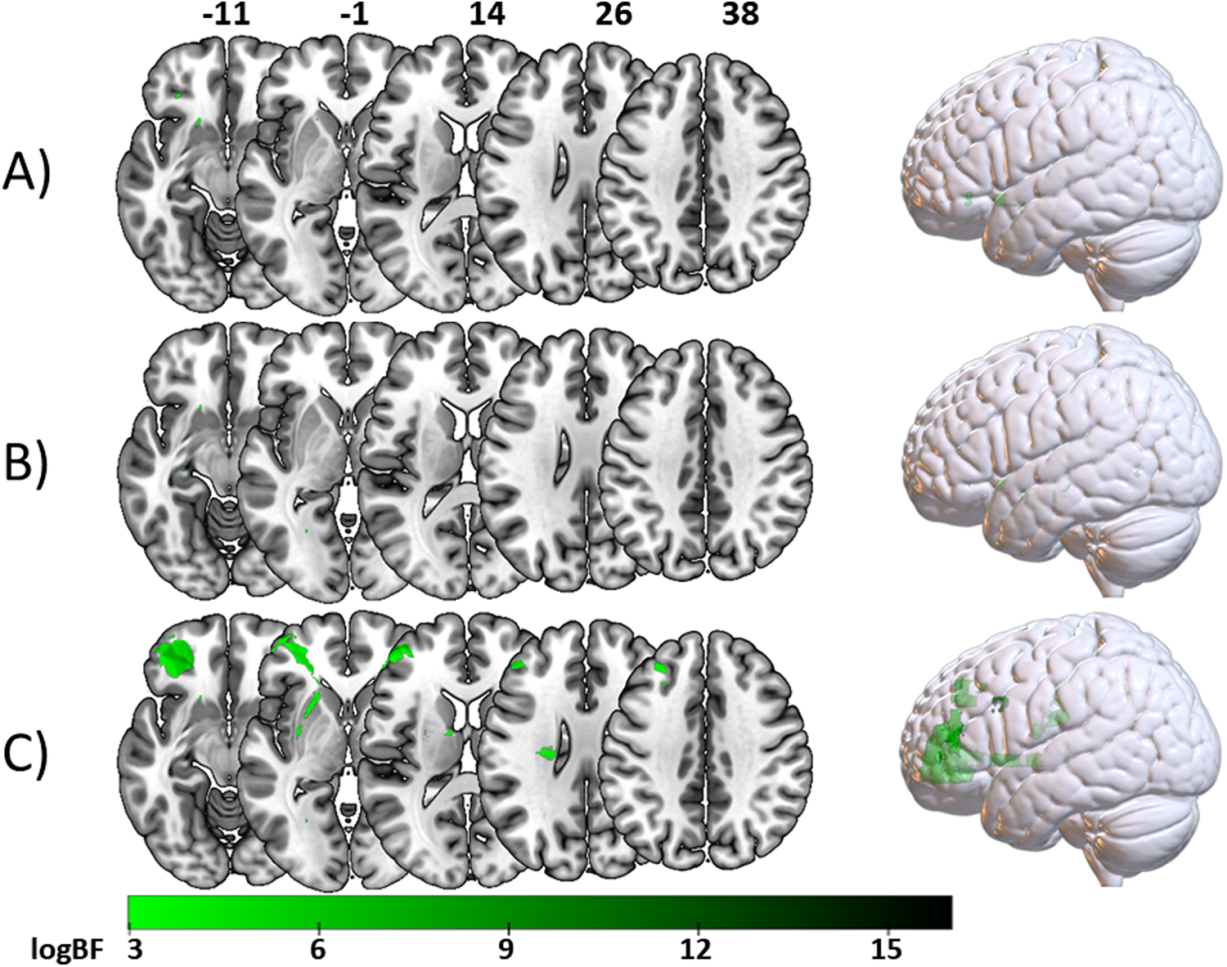
Bayesian associations between voxelwise brain damage and finger imitation deficits. Depicted are all voxels with a logBF > 2.99. Voxels resulting from the VLSM on (A) the ‘*full sample*’, (B) the ‘*shared finger sub-sample*’, and (C) the ‘*isolated sub-sample*’.

Applying the Bayesian GLM on the ‘*full sample*’ to uncover in the neural correlates of hand imitation skills led to 23842 voxels (BF_max_= 513,733.53). The same analysis on the ‘*shared hand sub-sample*’ led to 5639 voxels (BF_max_= 24,631.15). In the ‘*isolated sub-sample*’, 39442 voxels (BF_max_= 5,928,694.17) were observed. SeeFigure 2for voxels with sufficient evidence for an association between voxel and hand imitation deficit. All analyses uncovered a larger cluster around the borders of the occipital parietal and temporal lobe. Beyond, analyses on the ‘*full sample*’ and the ‘*shared hand sub-sample*’ showed a smaller frontal cluster. A comparison of results with and without lesion size control is provided inSupplementary Figure 1. Results with and without controlling for time post-stroke are compared inSupplementary Figure 2. Results from the frequentist analyses are reported inSupplementary Figure 3.

#### 3.1.2 Imitation of finger gestures

**Fig. 4.**
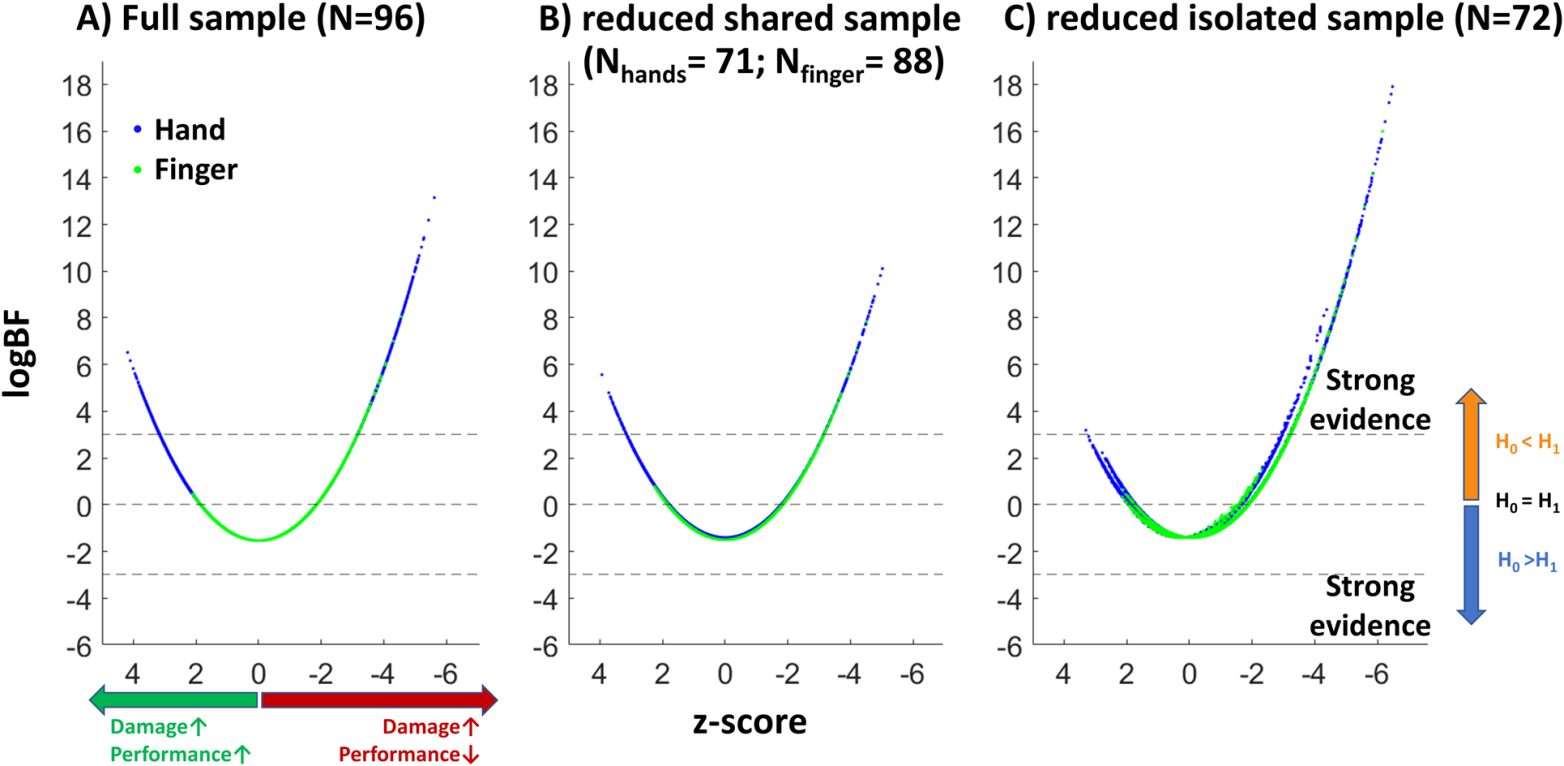
Scatter plots illustrating the relation of logBF and z-score for each analyzed voxel. Blue dots represent voxels resulting from analyses concerning hand imitation scores, and green dots indicate voxels resulting from analyses concerning finger imitation scores. (A) Contains all voxels from analyses on the ‘*full sample*’. (B) Represents the voxels derived from analyses on the ‘*shared hand sub-sample*and the ‘*shared finger sub-sample*’. (C) Depicts voxels found in analyses in the ‘*isolated sub-sample*’.

**Fig. 5.**
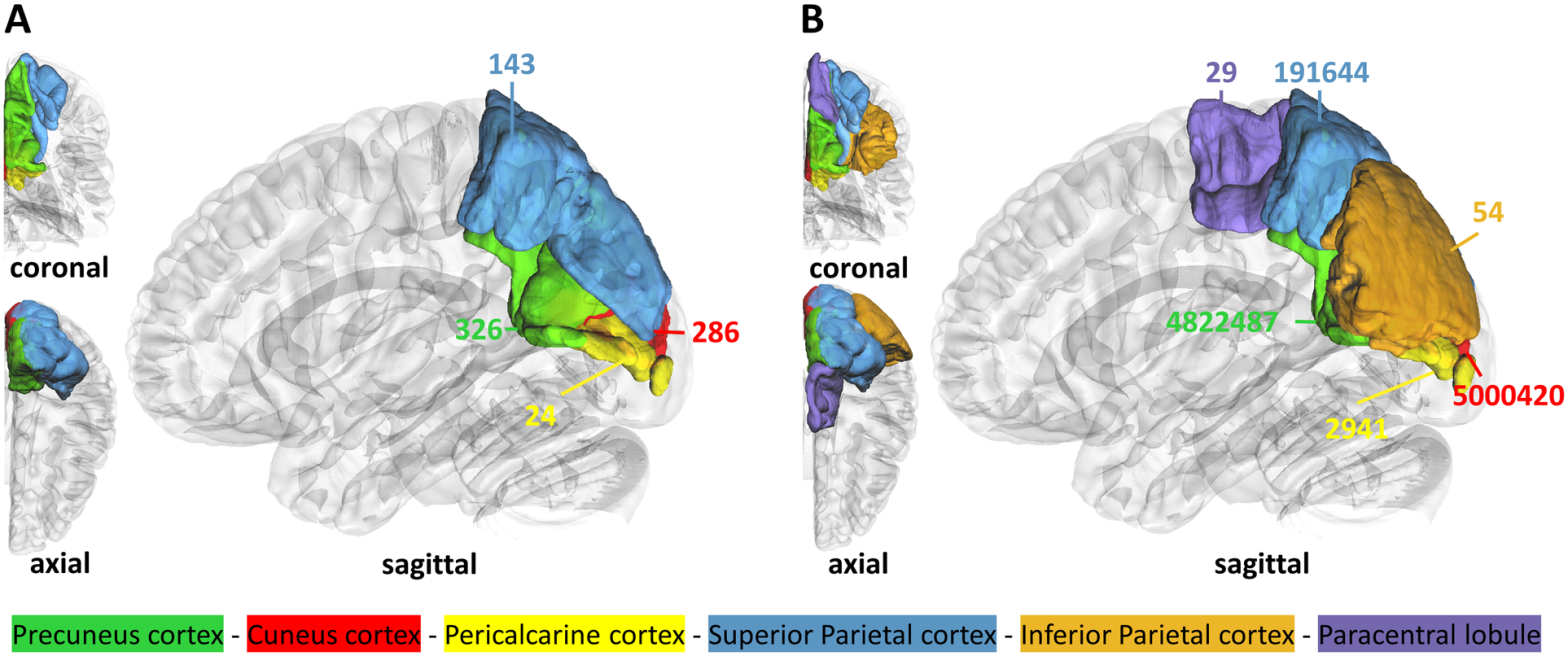
Left hemispheric brain regions with strong evidence against the null hypothesis concerning hand imitation deficits. (A) Results obtained from the ‘*full sample*’; (B) regions found in the ‘*isolated sub-sample*’. Numbers next to the coloured regions refer to the corresponding Bayes Factors.

**Fig. 6.**
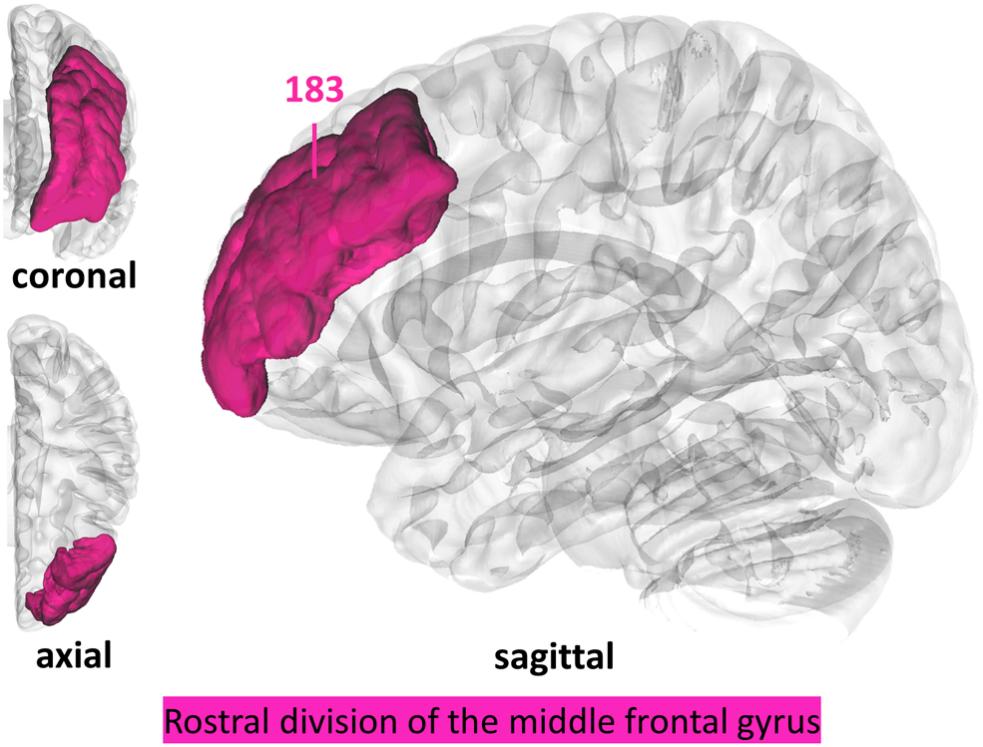
The rMFG in the left hemisphere showed strong evidence for an association with finger imitation deficits in our regionwise analysis testing for Bayesian lesion deficit inference in the ‘*isolated sub-sample*’.

In the ‘*full sample*’, the BLDI uncovered small anterior voxel clusters associated with the finger imitation score, containing 321 voxels in total (BF_max_= 3,127.50). For the ‘*shared finger sub-sample*’, 299 voxels in total (BF_max_= 2,544.70) were found. They formed minimal clusters both anteriorly and more posterior. In the ‘*isolated sub-sample*’, the Bayesian approach led to considerably larger voxel clusters containing 11373 voxels in total (BF_max_= 8,780,833.98), with a strong anterior focus in the pars orbitalis and the rostral division of the middle frontal gyrus (rMFG). Voxel clusters are visualized inFigure 3. A comparison of results with and without lesion size control is provided inSupplementary Figure 4. Results with and without controlling for time post stroke are compared inSupplementary Figure 5. Results from the frequentist analysis can be derived fromSupplementary Figure 6.

#### 3.1.3 logBFs in relation to z-scores

To have a direct comparison between Bayesian and frequentist statistics, that is logBFs, z-scores and their relation to one another, we created scatterplots for the statistical values resulting from analyses on the ‘*full sample*’ (seeFig. 4A), the ‘*shared hand sub-sample*and the*shared finger sub-sample*’ (seeFig. 4B), and the ‘*isolated sub-sample*’ (seeFig. 4C). All in all, the distributions resulting from analyses of the ‘*full sample*’, the ‘*shared hand sub-sample*and the*shared finger sub-sample*’ were skewed towards both lower z-scores and logBFs in reference to analyses on the ‘*isolated sub-sample*’. Another noteworthy observation is that a fraction of voxels with sufficient logBFs for an association with hand imitation in the ‘*full sample*’ and ‘*shared hand sub-sample*’ were found to have positive z-scores. That is, they showed a*reverse association*whereby voxel damage indicated a better performance. When locating these voxels, we found them to form the anterior clusters in the ‘*full sample*’ and ‘*shared hand sub-sample*’ (seeFig. 2A, Babove). In contrast, voxels with strong Bayesian evidence for the H1 in the ‘*isolated sub-sample*’ had almost exclusively negative z-scores representing damage leading to worse performance. For a better localization of voxels indicating sufficient evidence for an association, all results were overlapped with anatomical parcellations based on the atlas byDesikan et al. (2006). A table containing this information can be found inSupplementary Table 1.

### 3.2 Regionwise lesion-symptom mapping

Like in the voxelwise lesion symptom mapping, our hypotheses should also be investigated on the region-level. Analyses were conducted using the same dependent variables and samples, but now were used to model (continuous) regionwise- rather than (binary) voxelwise-brain damage. Scatterplots displaying regions’ logBFs in relation to their t-scores allow a more detailed insight into the sensitivity differences we assumed (see above). These scatterplots further allowed us to check for a potential positive bias for brain regions truly associated with hand imitation deficits in analysis of finger imitation deficit (and vice versa) in analyses on the ‘full sample’.

#### 3.2.1 Imitation of hand gestures

**Fig. 7.**
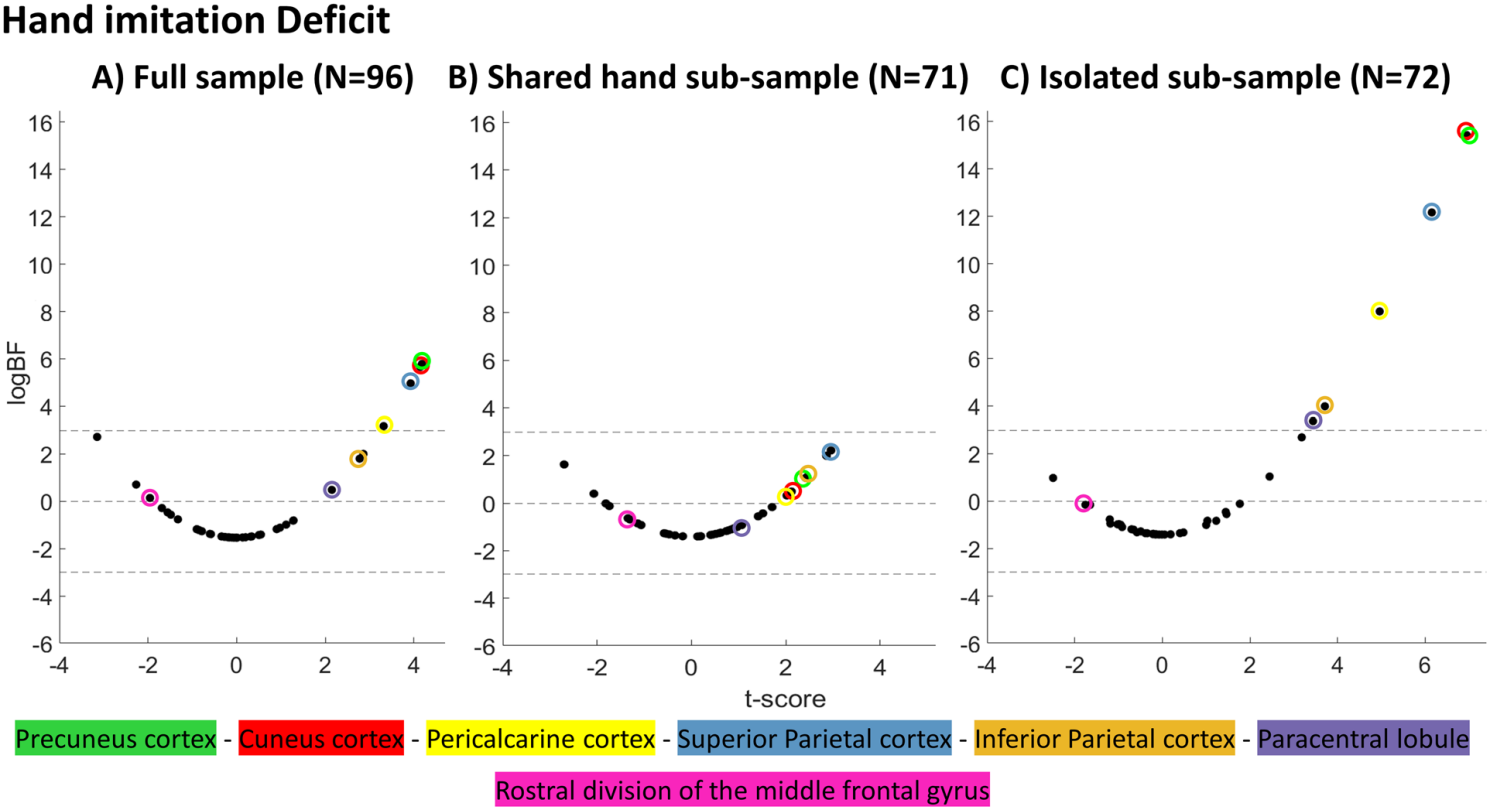
Scatter plots illustrating each region’s logBF relative to its t-score for the three conditions investigating the hand imitation deficit.

**Fig. 8.**
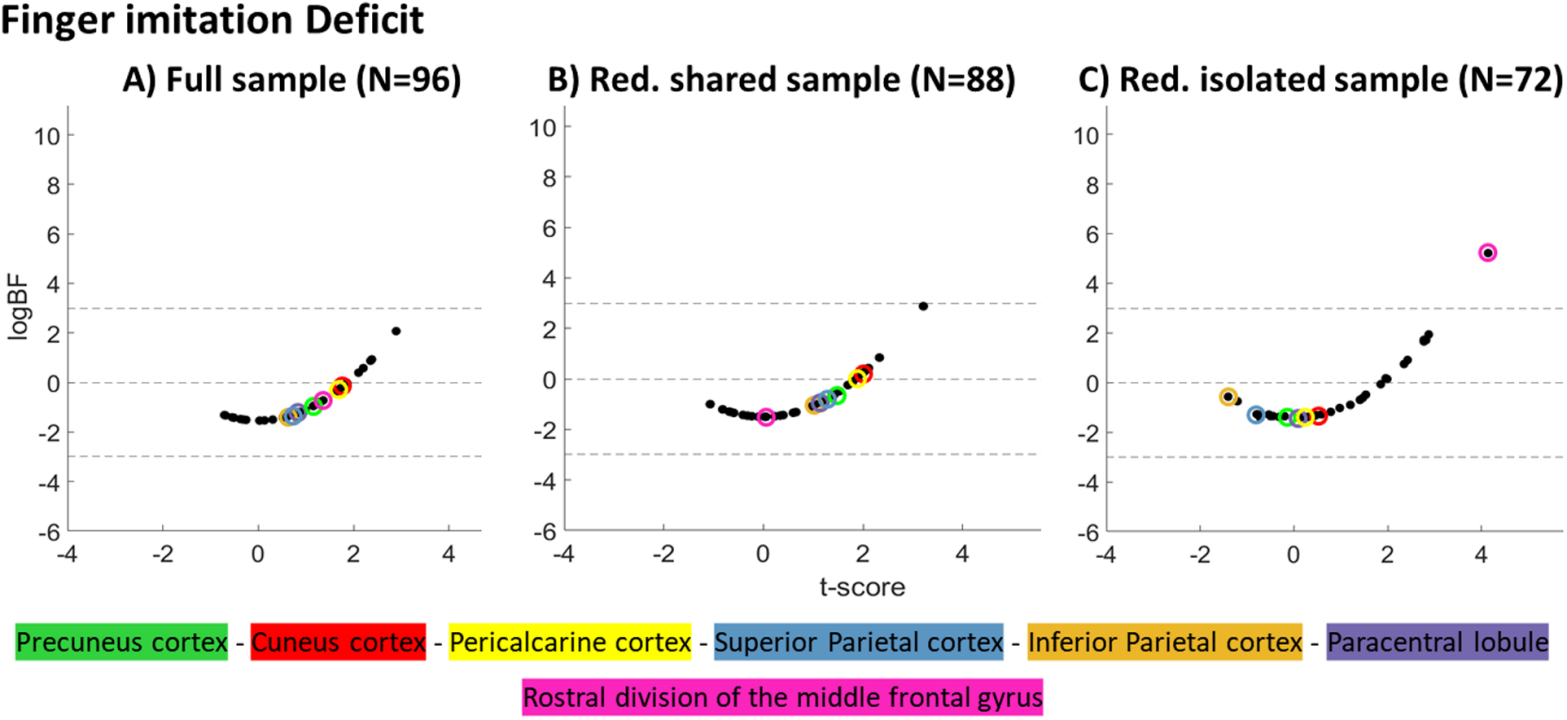
Scatter plots show regionwise logBFs relative to their t-scores for the three samples we investigated in context of deficits in finger imitation.

As with the VLSM, we report all regions with an evidence BF > 20. In the ‘*full sample*’, the regionwise Bayesian lesion-symptom mapping uncovered lesion-symptom associations with the strongest evidence against the null hypothesis between deficits in the imitation of hand gestures and the precuneus (BF = 326), followed by the cuneus (BF = 286), superior parietal lobe (BF = 143), and the pericalcarine cortex (BF = 24). There were no regions surpassing the critical BF in the ‘*shared hand sub-sample*’. The ‘*isolated sub-sample*’, in contrast, uncovered even more regions and showed overall higher BFs than the ‘*full sample*’. The regions with the strongest evidence against the H0 were the cuneus (BF = 5,000,420) and precuneus (BF = 4,822,487), superior parietal cortex (BF = 191,644), and the pericalcarine cortex (BF = 2,941), followed by the inferior parietal lobe (BF = 54) and paracentral lobule (BF = 29). Depictions of the brain regions found in the ‘*full sample*’ and ‘*isolated sub-sample*’ can be found inFigure 5. Results from frequentist analyses are illustrated inSupplementary Figure 7; results from the Bayesian approach with control for time since lesion are found inSupplementary Fig. 8.

#### 3.2.2 Imitation of finger gestures

Neither the Bayesian GLMs on the finger imitation scores of the ‘*full sample*’ nor the ‘*shared finger sub-sample*’ uncovered any region with a Bayes factor higher than 20. In the ‘*isolated sub-sample*’, the rMFG received a BF of 183 (seeFig. 6). Frequentist analyses did not result in any significant region-behavior associations.

#### 3.2.3 logBFs in relation to t-scores

Analyses on the ‘*isolated sub-sample*’ and hand scores boosted both logBFs and t-scores of regions that were found to be associated with a hand deficit in the ‘*full sample*’ and uncovered two additional regions with sufficient Bayesian evidence for a region-deficit association. With the exception of the paracentral lobule (Desikan et al., 2006), these regions were also among those with the highest logBFs in the ‘*shared hand sub-sample*’, albeit not surpassing the critical threshold. rMFG, which had been found in association with finger imitation deficits in the ‘*isolated sub-sample*’, persisted around zero regardless of the sample. Interestingly, t-values indicated a trend towards an inverse protective, rather than a damaging effect (for illustrations seeFig. 7). As can be depicted from the results on the finger imitation scores (Fig. 8), the rMFG, which demonstrated strong evidence for an association in the ‘*isolated sub-sample*’, was assigned a negative logBF in both other samples. On the contrary, regions associated with hand imitation had tendencies towards increased logBFs in the ‘*full sample*’ and ‘*shared hand sub-sample*’. A summary of all regions, their respective BFs logBFs and t-scores can be found inSupplementary Tables 2 and 3.

### 3.3 Control analyses with sample size of n = 8

While all in all Bayesian, in comparison with frequentist statistics, have advantages when analysing smaller samples (Sperber et al., 2023), the considerable size difference between patient groups with isolated hand imitation deficits (N = 25) and finger imitation deficits (N = 8) limits the comparability of the results. The smaller the group with a given deficit, the larger is the role of an individual patient in the association, which decreases the generalizability. Worst case, one might argue, that the rMFG represents a result obtained by chance, which could have emerged with an isolated hand deficit patient group of a similar size as well. We, therefore, created an additional 10,000 sub-samples in which we removed all but 8 randomly selected patients from the ‘*isolated sub-sample*’ with an isolated hand imitation deficit. Bayesian evidence for hand imitation skills was once more assessed in all those samples. We observed that, all regions found in the original analysis on hand imitation skills in the ‘*isolated sub-sample*’ co-occurred to varying degrees. In addition, our permutations uncovered the lingual and supramarginal gyrus in a considerable portion of analyses. In contrast, the rMFG was not uncovered by a single analysis (for detailed results seeTable 2andFig. 9).

**Table 2.** Absolute frequencies and statistical parameters for regions uncovered by the 10,000 control analyses.

Region	Permutations included	Permutations uncovered	Average BF (standard deviation)
Banks of the superior temporal sulcus	10,000	157	54.15 (75.54)
Caudate	10,000	3	34.47 (18.84)
**Cuneus**	**786**	**786**	**34211645979227.50** **(216371951922944)**
**Inferior parietal cortex**	**10,000**	**4,403**	**4323.89 (31289.84)**
Isthmus division of the cingulated	162	28	1450.20 (2667.58)
Lingual cortex	10,000	2,430	2106.74 (8136.21)
**Paracentral cortex**	**8,848**	**3,221**	**1365581397.27** **(45233279813.98)**
Parahippocampal gyrus	10,000	1	29.70 (0)
Pars opercularis of the inferior frontal gyrus	10,000	6	22.59 (1.51)
**Pericalcarine cortex**	**1,644**	**1,441**	**1998264942755.42** **(23739960215026.8)**
Postcentral gyrus	10,000	157	42.15 (12.59)
Posterior cingulate	10,000	22	33.15 (12.59)
**Precuneus**	**10,000**	**6,795**	**1531732073785320.00** **(37428619053823100.00)**
Rostral middle frontal	10,000	0	- (-)
**Superior parietal**	**10,000**	**8,427**	**961507228990.94** **(30415090503321.20)**
Supramarginal	10,000	3,670	3244.43 (24008.66)

Regions previously found in the analysis containing all patients with isolated hand imitation deficits are emphasized in bold.

**Fig. 9.**
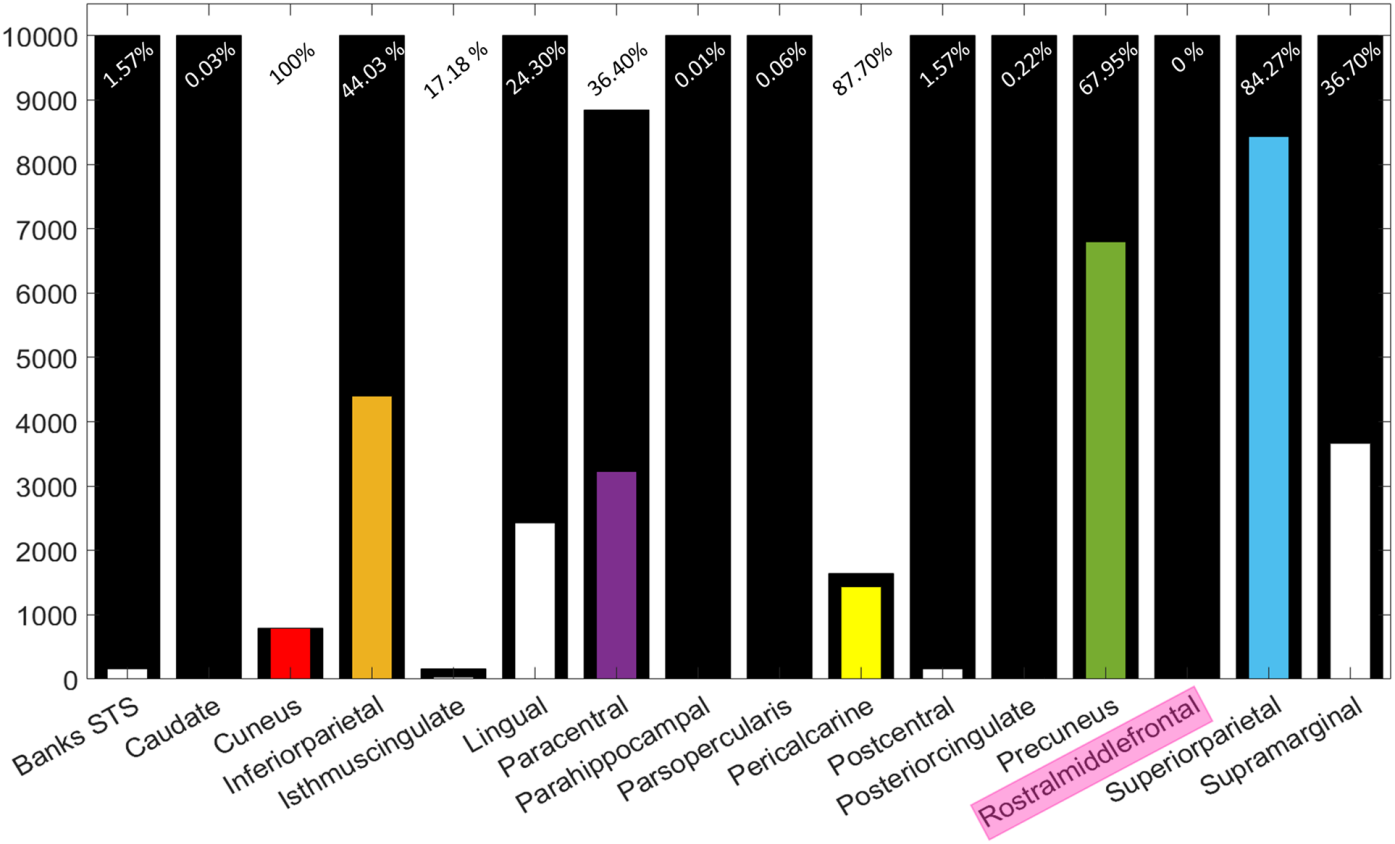
Regionwise associations with isolated hand imitation skills in the 10,000 control analyses. Black bars represent how often each region was included in the analyses. Colored bars represent regions uncovered in previous regionwise analyses. White bars represent regions exclusively found in the 10,000 control analyses.

## 4 Discussion

Our results support the notion of a neural dissociation of finger and hand imitation skills. Hand imitation deficits were found to be associated with damage to more posterior parts of the brain, while isolated finger imitation deficits were located more anteriorly. Isolated hand imitation deficits were associated with damage to mostly parietal and occipital structures. Isolated finger imitation deficits, in contrast, showed a focus in the frontal lobe. While we did uncover one frontal voxel cluster in association with hand imitation deficits, the comparison of frequentist and Bayesian parameters revealed that this cluster had protective rather than damaging properties, as indicated by the positive signs of the frequentist parameters. The dissociation proved stable with and without control for lesion size.

### 4.1 Neural dissociations between anatomical hand and finger representations

Inferior and superior parietal lobe proved to be the most solid brain-behavior associations concerning hand deficits. They were uncovered by both voxel and regionwise approaches whereby the superior parietal cortex demonstrated both a larger voxel cluster relative to its size (28.34% vs. 22.37%) in the voxelwise lesion-symptom mapping and an overall higher BFs in the regionwise approaches. This is in line with a large proportion of previous research, which reported one or both of these regions as hotspots in context with hand imitation deficits (Achilles et al., 2017; Dovern et al., 2011;Goldenberg & Karnath, 2006;Hoeren et al., 2014). The involvement of precuneus, cuneus, and pericalcarine cortex is, to our knowledge, observed for the first time in the context with hand imitation deficits in lesion symptom mapping. Interestingly, in the regionwise analysis approach, these regions showed an even stronger association than the inferior parietal lobe. It might be that cuneus and pericalcarine cortex, in particular, account for proprioceptive aspects of hand imitation deficits. Lesions to these areas have been found in context with deficits in kinesthetic matching, which required the ability to sense the position of once arm in space without visual feedback (Chilvers et al., 2021). It may well be that these regions represent similar proprioceptive deficits in our results. While our patients were able to see and visually control their finger gestures during the test, hand gestures required the hand to be positioned relative to the face and, therefore, outside the patient’s own visual field, that is, there was thus no possibility of visual control for hand gestures. Potential proprioceptive deficits could therefore be compensated in the finger but not the hand imitation tasks.

Previous studies stressed, in particular, the inferior frontal gyrus (IFG) and middle frontal gyrus (MFG) in context with finger imitation deficits (Goldenberg & Karnath, 2006;Haaland et al., 2000). Indeed, in our significant voxel cluster in the ‘*isolated sub-sample*’, 18.6 % of the pars orbitalis in the IFG was damaged, which made it the area with the greatest relative damage in this group in the voxelwise approach, followed by rMFG (12.4%). The latter was the only parcel uncovered in the regionwise approach. Thereby, our results revealed finger imitation even more anteriorly in the IFG than previous studies that found the center of lesioned mass more in the pars opercularis (Goldenberg & Karnath, 2006). Our results in the middle frontal gyrus closely resemble previous accounts byHaaland et al. (2000). They found disproportionally frequent damage to Broadman areas 9 and 46 in patients with impaired finger gestures imitation. Both areas reported byHaaland et al. (2000)show considerable overlap with the region defined as rMFG byDesikan et al. (2006). Accounts for a frontal lesion focus for finger imitation deficits are not limited to the left, but reported also for the right hemisphere, where the frequency of finger imitation deficits is overall higher (Goldenberg, 1999). To our knowledge, these brain behavior associations have been less controversial than their counterparts in the left hemisphere. It might be that disconnections to right hemispheric frontal areas via the corpus callosum contribute to isolated finger deficits after left hemispheric frontal damage. When combined with a white matter streamline atlas (Yeh et al., 2018), the rMFG (Desikan et al., 2006) does, indeed, show a dense structural connection to frontal regions in the opposite hemisphere. This research question requires a more thorough investigation in future works.

Our control analyses, in which 10,000 sub-samples were formed, each with 8 patients from the ‘*isolated sub-sample*’ with an isolated hand imitation deficit, yielded interesting results. First, we observed that regions, which surpassed our Bayesian cut-off (BF > 20) in the original analyses on hand imitation skills in the ‘*isolated sub-sample*’, obtained (on average) larger BFs than those regions found exclusively in the control analyses (seeTable 2above). Also, the lingual gyrus, the isthmus division of the cingulate gyrus, (Desikan et al., 2006), and the supramarginal gyrus were uncovered at moderate rates and had considerable average BFs as well. This indicates that these regions—in addition to those areas found by the analysis on the ‘*isolated hand sub-sample*’—might play a role for the imitation of hand gestures. Importantly, all three of these additional regions are also located directly adjacent to at least one region we uncovered in the analyses on hand imitation skills in the original ‘*isolated sub-sample*’. This indicates that, alternatively, these regions might represent a localization-induced bias which took effect in the control analyses due to their decreased group size.

The rMFG, which was uncovered in the analysis investigating finger imitation scores in the ‘*isolated sub-sample*’, had a BF of 180, which is considerably higher than all those of regions, which we can confidently identify as unbiased false positives, that is, the caudate, parahippocampal gyrus, and pars opercularis of the IFG. Despite their overall lower BFs, the banks of the superior temporal sulcus, the postcentral gyrus, and the posterior cingulate (like the lingual gyrus, the isthmus division of the cingulate gyrus, and the supramarginal gyrus) are located directly adjacent to some of the original regions we found in the analyses on hand imitation skills in the original ‘*isolated sub-sample*’. Since the rMFG was found without any other accompanying regions, it further seems rather unlikely that the rMFG’s high BF represents a systematic bias caused by true positives in its proximity.

### 4.2 Sampling-induced biases

By using the scatterplots illustrating the relation of Bayesian and frequentist lesion mapping results (whichAchilles et al. (2017)created only for their voxelwise results) also for regionwise brain-behavior associations, we were able to observe the effects of the different sub-sampling conditions on specific regions. All results support the notion that analyses on the ‘*full sample*’ were biased by diluting effects caused by a joint analysis of patients with shared and isolated deficits. The basic principles underlying this mechanism are described by the partial injury problem (cf.Kinkingnéhun et al., 2007;Rorden et al., 2009). In general, regions associated with the relatively larger sub-group (i.e., shared vs. isolated deficit) have a better chance to prevail, while regions from the smaller group will be relatively more watered down, the larger the sample size difference is. Here, the resulting diluted association will be smaller, the smaller the true association of the voxel with the behavior of interest in the sample initially was. Despite a reduced sample size, analyses on our ‘*isolated sub-sample*’ consistently uncovered more and overall stronger brain-behavior associations than those on the ‘*full sample*’ containing both shared and isolated imitation deficits.

The inferior parietal cortex and the paracentral lobule were associated with isolated hand deficits but did not persist in our analyses on the ‘*full sample*’. Despite the removal of patients with isolated hand deficits, most isolated hand imitation areas remained among those with the strongest evidence for an association also in the ‘*shared hand sub-sample*’. Unfortunately, while patients with isolated deficits can be easily distinguished behaviorally, and it seems safe to assume that a patient with an isolated hand deficit will not have suffered damage to a*shared*or*isolated finger*area, the opposite conclusion for a shared imitation deficit cannot be made. There is no easy way to tell if a shared imitation deficit was caused by damage to anatomically*shared areas*, damage to both*isolated hand*and*finger areas*or even a mix of*shared*and*isolated areas*. However, high BFs for*isolated hand areas*in the ‘*shared hand sub-sample*’ indicate that lesions leading to a shared deficit often extend to*isolated hand areas*.

Concerning finger imitation deficits, the ‘*isolated sub-sample*’, uncovered associations with the rMFG. In the ‘*full sample*’, consisting of both patients with isolated and shared finger deficits, the rMFG showed a negative logBF, however, not as low as in the ‘*shared finger sub-sample*’ where the rMFG had one of the most negative logBFs. This observation indicates that, unlike*isolated hand areas*,*isolated finger areas*rarely suffer damage in patients with a shared imitation deficit, thereby challenging the notion that simultaneous damage to*isolated hand*and*finger areas*might lead to shared imitation deficits.

In particular, patients with a shared imitation deficit should drive the anticipated positive bias for areas truly associated with hand imitation deficits in analyses on finger deficits (and vice versa), since only their behavioral score can be wrongly associated to areas truly responsible for the hand imitation deficit. Patients with a shared deficit showed frequent involvement of*isolated hand areas*so that the bias could be nicely demonstrated for*isolated hand areas*in analyses on finger imitation scores. In the ‘*isolated sub-sample*’, patients with a shared deficit were excluded. That is, patients with a finger imitation deficit in this sample did not have accompanying damage to*isolated hand areas*. They were among those areas with the strongest tendencies against the H1 according to their logBFs or even suggested*reverse associations*according to their t-score. In the ‘*full sample*’, the hypothesized bias was mitigated by the presence of isolated hand deficit patients, who had lesions in*isolated hand areas*without a finger deficit. In the ‘*shared finger sub-sample*’, those very patients were excluded, which led the bias to be particularly strong. Cuneus cortex and pericalcarine cortex here even switched sign and became positive (seeFig. 8above).

Our results indicate that, in contrast to*isolated hand areas*,*isolated finger areas*were rarely damaged in patients with a shared deficit. For this very reason, the rMFG with isolated finger imitation did not bias the analyses on hand imitation skills, as the*isolated hand areas*did with the analyses on finger imitation skills. The bias here would not be expected to begin with, since an area damaged only in the isolated deficit cannot be misattributed to the respective other deficit. Indeed, the logBFs of the rMFG persisted around zero, indicating equal evidence for and against the H1 while frequentist parameters indicated a trend towards reverse effects regardless of the sample. This result contests the idea that simultaneous damage to isolated hand and finger areas is responsible for shared imitation deficits.

While we did not uncover any*shared areas*, with sufficient Bayesian evidence, the absence of damage to*isolated finger areas*in patients with a shared deficit indirectly supports their existence. With a BF of 17, also the lingual gyrus was approaching the Bayesian cut-off that we adapted fromAchilles et al. (2017)in the ‘*shared finger sub-sample*’; it was among the highest BFs also in the ‘*shared hand sub-sample*’ and when it came to associations of both hand and finger deficits in analyses on the ‘*full sample*’ (ranging from BFs of 7.4 to 7.9), but had considerably lower BFs in the ‘*isolated sub-samples*’ (seeSupplementary Table 2). Its posterior location could offer a reasonable explanation why patients with a shared deficit frequently suffered co-occurring damage of*isolated hand areas*, which are in the close proximity to the lingual gyrus, while damage appeared to rarely extend to the more distant*isolated finger areas*. Indeed, the lingual gyrus has previously been found in a functional imaging study, when participants imitated actions from a third-person but not from a first-person perspective (Jackson et al., 2006). The lingual gyrus was, therefore, suggested to potentially contribute to visuospatial transformation necessary for the former but not for the latter (Jackson et al., 2006). Without the ability to transform observed gestures modelled by someone else onto one’s own body, imitation should fail regardless of the body part to be imitated, which would explain why it affects both hand and finger imitation.

Another reason why we did not uncover a specific grey matter region might be that a shared deficit could result from damage to white matter fibers connecting the isolated hand and finger regions, rather than from damage to a designated grey matter area. Indeed, the voxel cluster associated with a shared finger imitation deficit after control for time since stroke (i.e., the only analysis that resulted in a somewhat interpretable cluster) consisted primarily of voxels in the white matter (seeSupplementary Fig. 5B). When we used fiber tracking using a normative tractogram (Yeh et al., 2018), we uncovered that these voxels, indeed, contain numerous fibers that connect the finger area to some of the hand areas. In particular, inferior fronto-occipital fasiculus fibers connecting the rMFG with the inferior parietal lobe would be heavily affected by damage to the voxel cluster (seeSupplementary Table 4andSupplementary Fig. 8). Both structural and functional disconnection approaches appear to allow more complex anatomical inferences on the network level (Boes et al., 2015;Griffis et al., 2019). However, because this investigation aimed to evaluate previous analysis methods that did not account for structural or functional disconnection, we also refrained from using those approaches.

### 4.3 Reflections on the work of Achilles et al. (2017)

In contrast toAchilles et al. (2017), our results did not uncover any voxels surpassing the threshold for evidence for the H0; this might be attributable to our smaller sample size. Bayesian statistics quantify not only evidence for the H1 but also against it. But they do so at different rates, with those in favor of the H1 accumulating considerably faster (Johnson & Rossell, 2010). It has been argued that this asymmetry is unproblematic and simply reflects the fact that support for the absence of something is harder to quantify than support for its presence (van Ravenzwaaij & Wagenmakers, 2022). However, investigating evidence for the H0 will consequently require larger sample sizes than evidence for the H1.

In their regionwise lesion-symptom mapping,Achilles et al. (2017)uncovered an almost perfect overlay of regions with sufficient evidence for finger and hand imitation deficits (albeit with variations in the size of the evidence). We did not replicate these results in our analyses of the ‘*full sample*’*.*However, two effects which we uncovered and which are elaborated in the following offer potential explanations how these overlays were fabricated and how*isolated hand*and*finger areas*might have been covered up in their results.

The first effect is directly related to our hypothesis, which assumed a bias introduced by patients with a shared deficit. Bayesian approaches are known to be more liberal than their frequentist counterparts, which is of particular advantage in situations with limited power but, in turn, increases the risk of false positives (Sperber et al., 2023). In a recent simulation study on the voxel level and a sample size closely resembling the one byAchilles et al. (2017), the major part of (on average ~11,600) true positives was uncovered, but at the same time one third of all (on average ~748,000) true negatives were attested a wrongful association (Sperber et al., 2023). While these numbers cannot be directly applied on real data, they offer a rough estimate on one of the largest drawbacks of Bayesian approaches. As hypothesized, the analyses of finger imitation deficits on the ‘*full sample*’ and ‘*shared finger sub-sample*’ (i.e., those containing a large number of patients with a shared deficit) produced a systematic bias for regions truly associated with hand imitation deficits (cf.Fig. 8: the cuneus and pericalcarine cortex were assigned a stronger association to finger imitation than the rMFG, which was the only area uncovered in the ‘*isolated sub-sample*’). Apparently overlapping results from an undivided sample, like the one byAchilles et al. (2017)could, therefore, represent true associations of the overrepresented hand imitation, which also emerged (as false positives) in the analysis on finger imitation deficits, while the*isolated finger areas*might have been diluted to the point where they are not detected anywhere. Our ‘*full sample*’ was both smaller, and had a higher ratio of isolated finger deficits (8:96) than the one fromAchilles et al. (2017; 10:257), which caused a lower false positive risk and overall underrepresentation of*isolated finger areas*in our case. This explains why, in contrast to the effects byAchilles et al. (2017), ours were only visible in lower BF ranges and did not alter results above our threshold for meaningful evidence.

The second effect emerged from the dispersions of Bayesian and frequentist statistical parameters in our results. Shared neural correlates for hand and finger imitation might have, in fact, represented opposite associations. WhileAchilles et al. (2017)offered insight into the ratios between logBFs and t-scores in their voxelwise approach, regionwise results are reported exclusively for Bayesian analyses. Bayesian statistics offer a quantification of evidence, not only against but also for the H0, however, a clear drawback is that no information can be provided concerning the direction of the uncovered effects. Our VLSMs on hand imitation deficits offer a concrete example of how Bayesian evidence can uncover both the expected negative and reverse positive associations. It is, therefore, possible that one and the same region is uncovered due to a negative association in one analysis and due to a positive association in the other. Without the combined use of Bayesian and frequentist approaches, there is no way to distinguish them.

LikeHoeren et al. (2014)before them,Achilles et al. (2017)generated a difference-score for each patient by subtracting the finger imitation score from the hand imitation score, in addition to their analyses on pure hand and finger imitation scores,. While this measure does, indeed, estimate the behavioural difference between patients’ hand and finger imitation skills, it is not an appropriate means to provide meaningful brain-behavior associations which is the reason why we did not (re)analyze this score in the present study. This score potentially assigns patients with no deficit at all (e.g., 20–18 points), a deficit in both (e.g., 8–6 points), and an isolated deficit in one imitation type (e.g., 18–16 points), an equal difference-score of two. While it does offer an estimate of the extent of one behavioral deficit relative to the other, we would not expect neural similarity among those three patients. Lesion symptom mapping based on the difference score would therefore aim at identifying neural similarities of fundamentally different behaviour combinations. Even with a sound sampling approach, results from this analysis cannot be considered as evidence for a task dissociation.

## 5 Conclusion

The present investigations support the notion of a posterior-anterior anatomo-behavioral dissociation for hand and finger imitation skills. It further raises the question whether, in addition, the lingual gyrus plays a role in (shared) deficits of both hand and finger imitation skills, which should be further investigated in future studies. Beyond, the present results demonstrate that certain sampling approaches can dilute existing associations representing isolated deficits of one imitation skill but not the other. They also show that (and how, in combination with the association problem) such sampling approaches can lead to misleading co-associations between hand and finger imitation deficits. Finally, it shows the exclusive use of Bayesian approaches might uncover opposing associations, which cannot be controlled for. Importantly, both issues can be countered: the former by appropriate sub-sampling, the latter by additional frequentist analyses.

## Supplementary Material

Supplementary Material

## Data Availability

While public archiving of anonymized patient data is prevented by the ethics approval, access to the data can be provided upon reasonable request by Georg Goldenberg. The code used for data pre- and post-processing can be requested from the first author.
