# Supplementary material for: Bayesian evidence for the neural dissociation between finger and hand imitation skills
Source: Imaging Neurosci (Camb). 2024 Nov 1;2:imag-2-00342. doi: 10.1162/imag_a_00342 (PMC12290844; doi:10.1162/imag_a_00342)
Supplement: Supplementary Material [file imag_a_00342-supp.pdf]

Supplementary materials

Bayesian evidence for the neural dissociation between finger and hand imitation skills

Hannah Rosenzopf, Lisa Röhrig, Georg Goldenberg, Hans-Otto Karnath

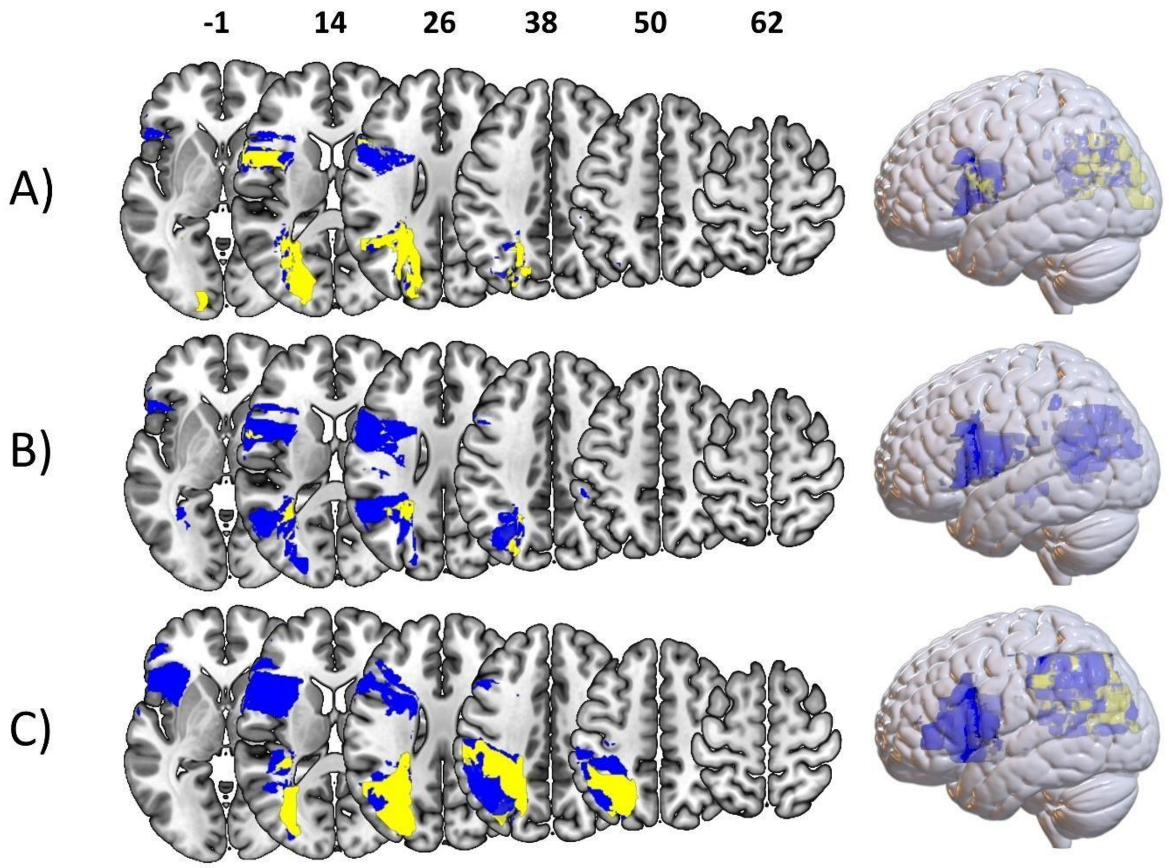

Supplementary figure 1: Comparison of results derived from the same analyses with vs. without lesion size control on hand imitation scores. Blue represents voxels with sufficient evidence for the H1 with lesion size control only, there were no voxels with sufficient evidence for the H1 without lesion size control only. Yellow voxels mark voxels uncovered by both analyses. A) Displays results from analyses on the full sample. B) Showcases voxels derived from analyses on the shared sub-sample. C) Represents voxels uncovered in the isolated subsample. Importantly, the frontal cluster that was found

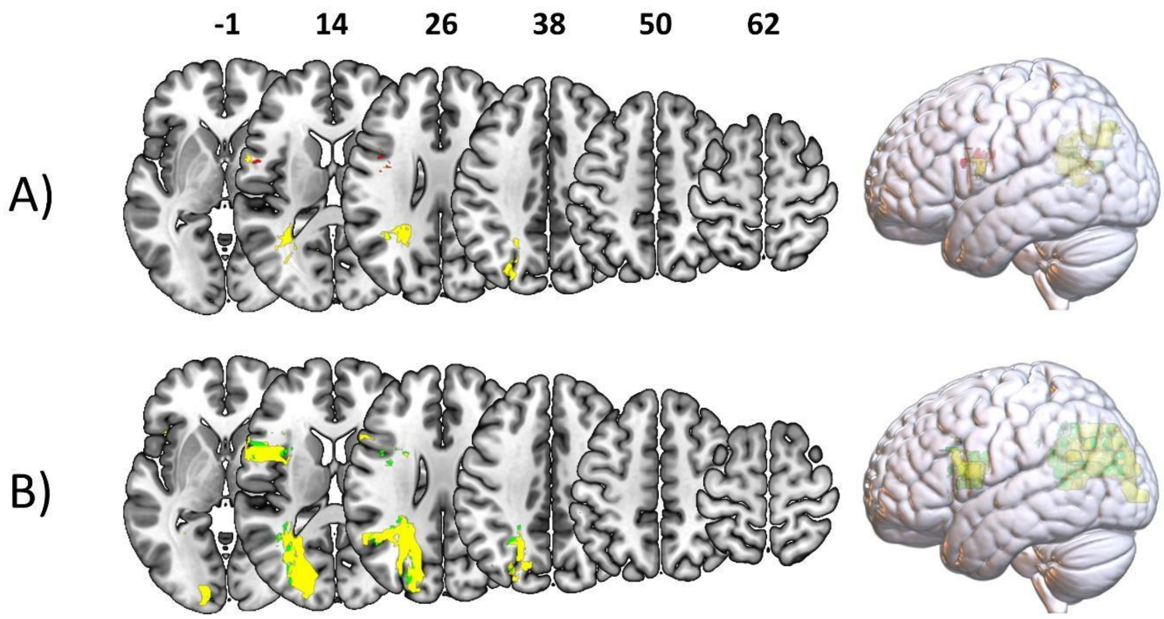

Supplementary figure 2: Comparison of results derived from the same analyses with vs. without control for time since stroke for hand imitation scores. Green represents voxels with sufficient evidence for the H1 with control for time since lesion only, there were no voxels with sufficient evidence for the H1 without control for time since lesion only. Yellow voxels mark voxels uncovered by both analyses. A) Displays results from analyses on the full sample. B) Showcases voxels derived from analyses on the shared sub-sample.

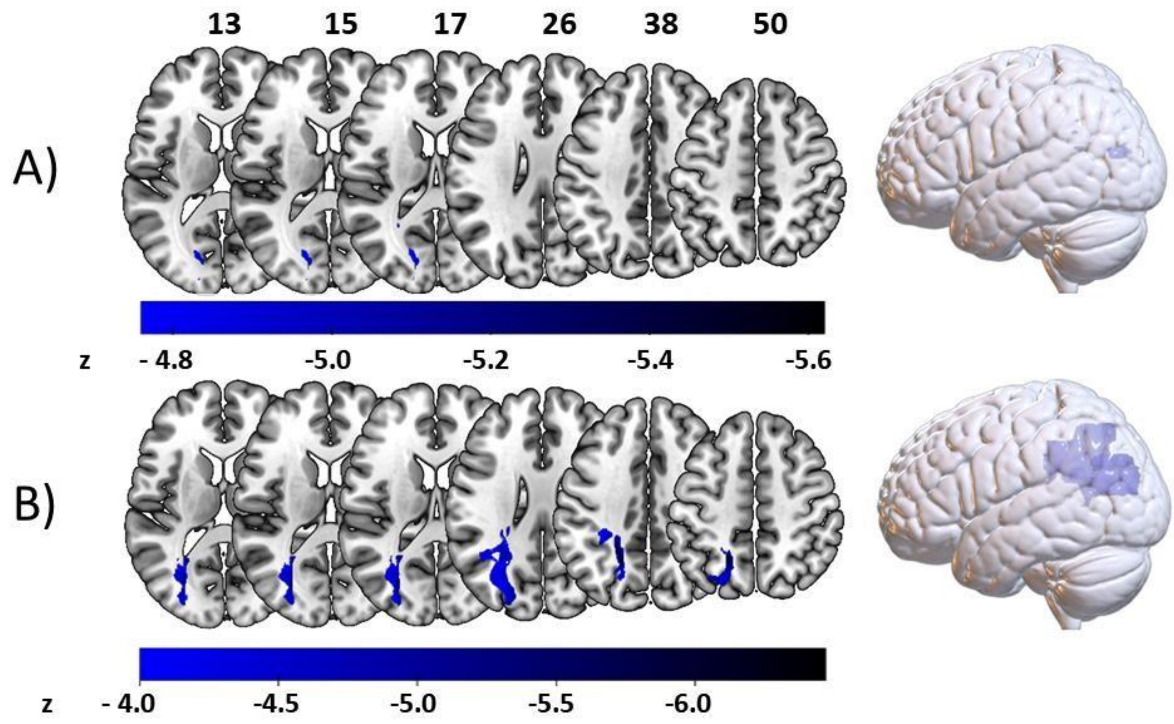

Supplementary figure 3: Frequentist associations between voxelwise brain damage and hand imitation deficit. A) Significant voxels resulting from the VLSM on the full sample uncovered a small posterior voxel cluster of 604 voxels (z_{crit}=-4.76; z_{max}=-5.61). B) The reduced isolated sample uncovered a larger posterior voxel cluster containing 13633 voxels (z_{crit}=-4.04; z_{max}=-6.47). There were no voxels with sufficient evidence in the reduced shared sample.

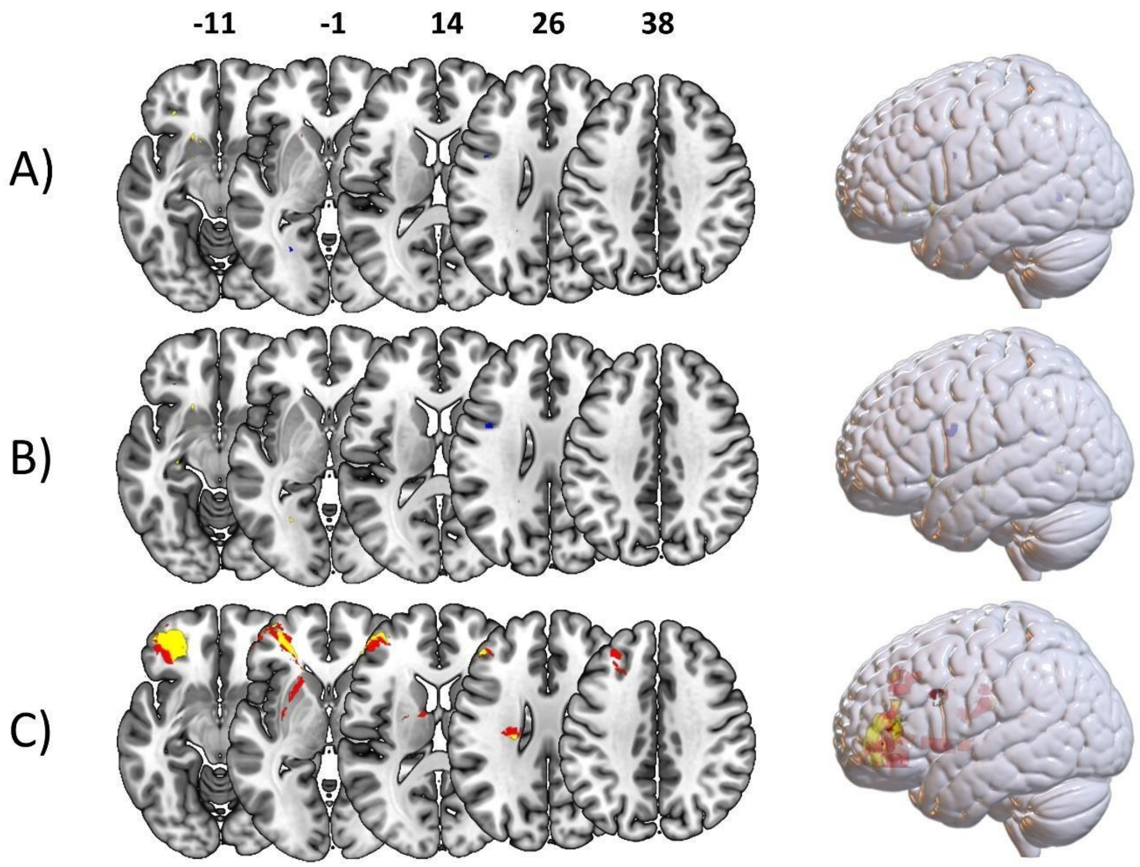

Supplementary figure 4: Comparison of results derived from the same analyses with vs. without lesion size control on finger imitation scores. Blue represents voxels with sufficient evidence for the H1 with lesion size control only, red without lesion size control only. Yellow voxels were uncovered by both analyses. Results are displayed from analyses on the A) full sample, B) shared sub-sample, and C) isolated sub-sample.

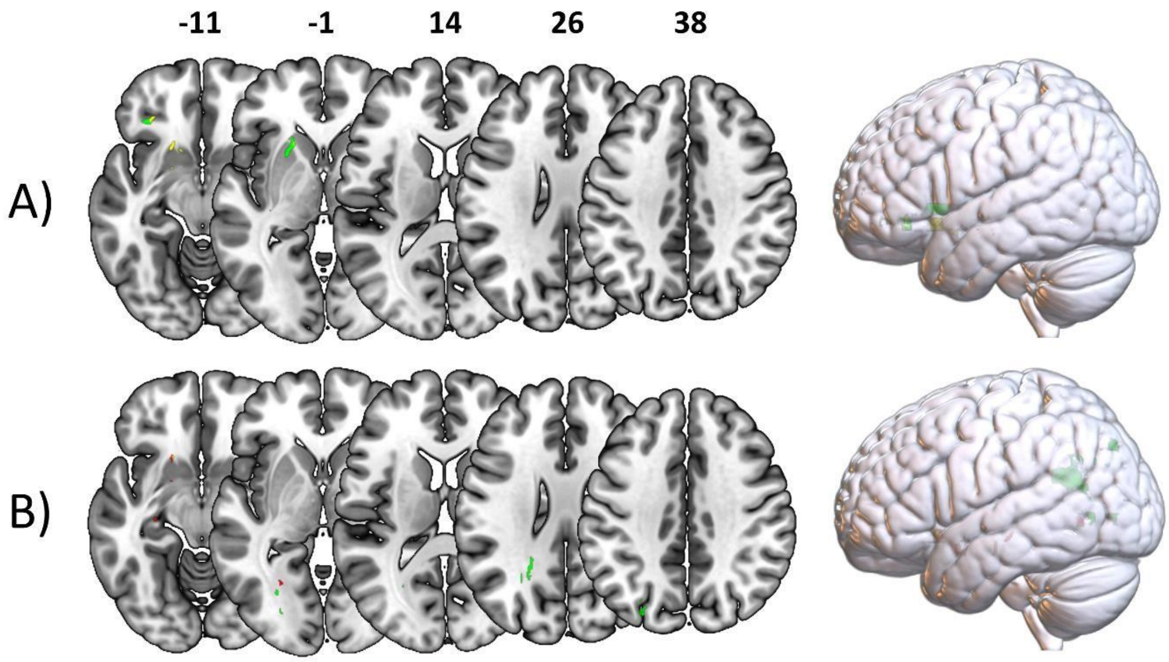

Supplementary figure 5: Comparison of results derived from the same analyses with vs. without control for time since stroke on finger imitation scores. Green represents voxels with sufficient evidence for the H1 with control for time since lesion only, red control only. Yellow voxels were uncovered by both analyses. Results are displayed from analyses on the A) full sample and B) shared sub-sample.

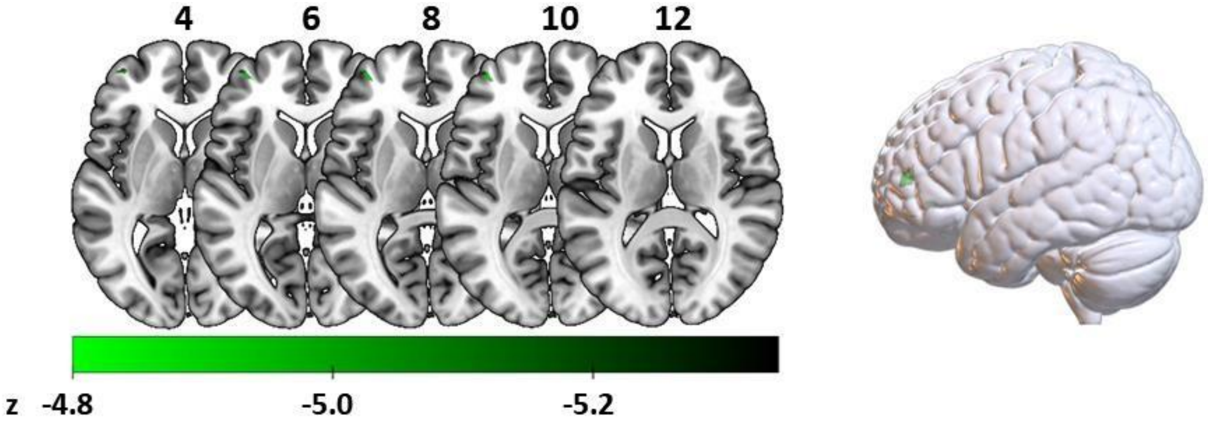

Supplementary figure 6: The Frequentist VLSM on finger imitation deficits produced significant results only in the reduced isolated sample, here 353 voxels were significant (z_{crit}=-

4.83; z_{max}=-6.16). Note that for the same VLSM including lesion size control, no significant voxels were found.

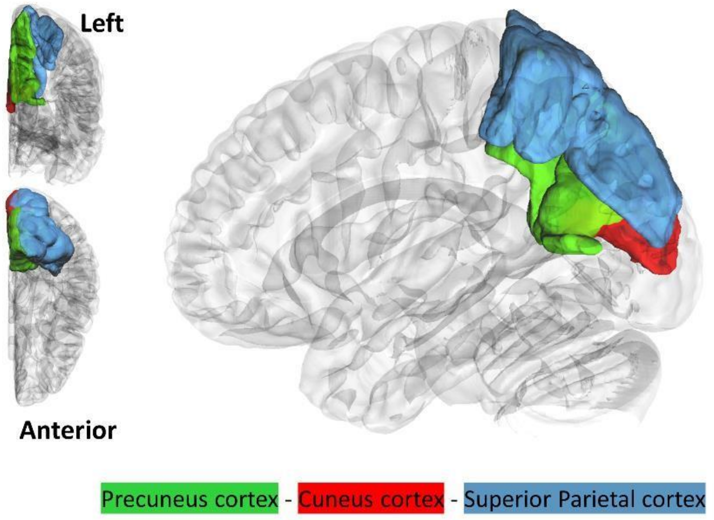

Supplementary Figure 7: Frequentist regionwise lesion-symptom mapping, based on the atlas parcellation by Desikan and colleagues (2006) uncovered significant results in only one of the analyses, namely, the analysis of hand imitation skills using the reduced isolated sample. Here, three regions surpassed the critical t-value of 5.64.

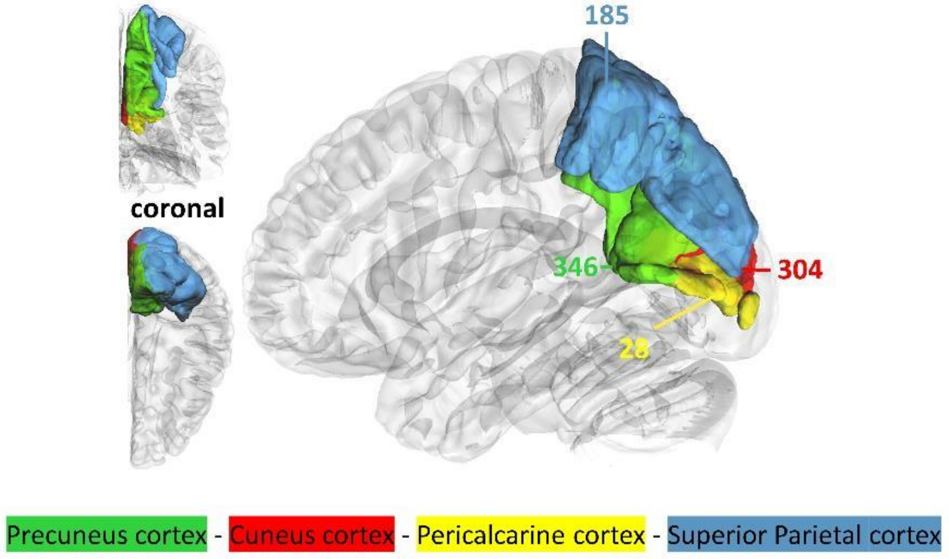

Supplementary Figure 8: Left hemispheric brain regions with strong evidence against the null hypothesis concerning hand imitation deficits using the Bayesian approach and control for time since lesion in the 'full sample'. In the 'shared hand sub-sample' did not uncover regions with sufficient evidence for an association

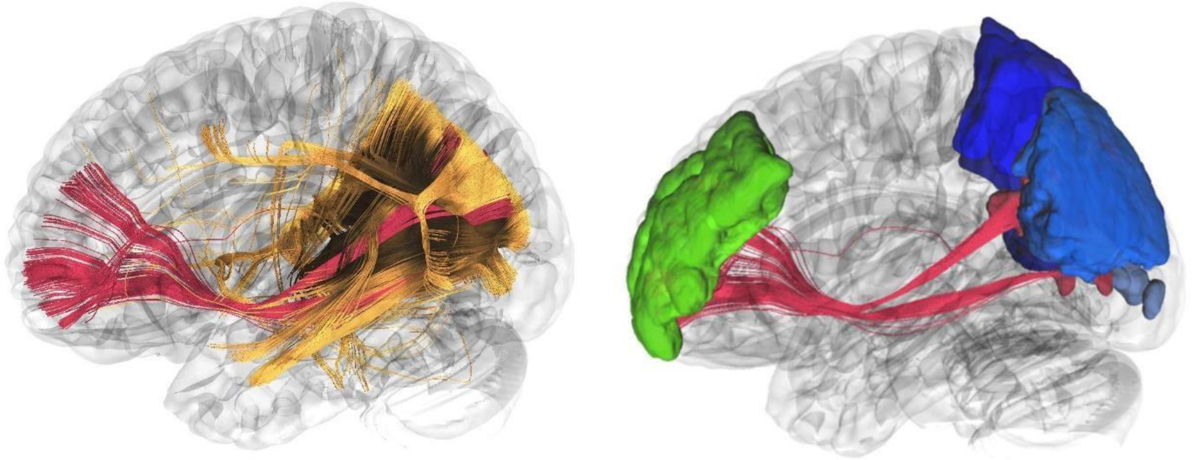

Supplementary figure 9: Disconnection profile derived from voxels that showed sufficient Bayesian evidence for an association with shared hand imitation deficits after controlling for time since stroke. A) An illustration of fibers intersecting with the voxel cluster shown in supplementary figure 5 B. Fibers connecting the finger area (rMFG) with one of the hand areas are coloured in red, connections between other regions are coloured in orange. B) showcases only the fibers disconnected between hand areas and the finger area as well as the respective areas (finger area green, hand areas blue, lesion cluster red).

Supplementary Table 1: Regionwise overlaps of voxels with sufficient Bayesian evidence for results from all sub-samples. abs = absolute numbers of voxels, black cells with white font mark those voxels with sufficient Bayesian evidence, but frequentist parameters indicating a *reverse association*.

		Hand all		Hand shared		Hand exclusive		Finger All		Finger shared		Finger exclusive	
Lobe	Label	abs	%	abs	%	abs	%	abs	%	abs	%	abs	%
occipital	Lateraloccipital	45	0.37	3	0.02	19	0.16	0	0.00	2	0.02	0	0.00
occipital	Cuneus	595	9.99	0	0.00	0	0.00	0	0.00	0	0.00	0	0.00
occipital	Pericalcarine	336	9.29	0	0.00	33	0.91	0	0.00	0	0.00	0	0.00
occipital	Lingual	8	0.06	0	0.00	0	0.00	0	0.00	19	0.15	0	0.00
parietal	Superiorparietal	1977	10.56	267	1.43	5304	28.34	0	0.00	0	0.00	0	0.00
parietal	Inferiorparietal	1549	6.57	535	2.27	5277	22.37	0	0.00	0	0.00	0	0.00
parietal	Precuneus	1831	10.31	28	0.16	142	0.80	0	0.00	0	0.00	0	0.00
parietal	Supramarginal	60	0.34	27	0.15	2731	15.47	0	0.00	0	0.00	0	0.00
parietal	Postcentral	0	0.00	0	0.00	505	3.45	0	0.00	0	0.00	0	0.00
temporal	Hippocampus	0	0.00	30	0.40	0	0.00	0	0.00	49	0.66	0	0.00
temporal	Banks STS	8	0.15	5	0.09	58	1.05	0	0.00	0	0.00	0	0.00
temporal	Superiortemporal	0	0.00	0	0.00	100	0.50	0	0.00	0	0.00	0	0.00
insular	Insula	181	1.36	21	0.16	0	0.00	0	0.00	6	0.05	5	0.04
subcortical	Amygdala	0	0.00	0	0.00	0	0.00	1	0.03	2	0.07	8	0.27
subcortical	Thalamus-Proper	0	0.00	0	0.00	0	0.00	0	0.00	0	0.00	20	0.24
subcortical	Accumbens-area	0	0.00	0	0.00	0	0.00	13	1.95	0	0.00	0	0.00
subcortical	Caudate'	0	0.00	0	0.00	0	0.00	33	0.57	13	0.23	138	2.40
subcortical	Putamen'	0	0.00	0	0.00	0	0.00	81	1.27	44	0.69	512	8.00
frontal	Precentral	798	4.14	286	1.48	8	0.04	0	0.00	0	0.00	3	0.02
frontal	Caudalmiddlefrontal	11	0.11	0	0.00	0	0.00	0	0.00	0	0.00	6	0.06
frontal	Parsopercularis	787	10.30	119	1.56	0	0.00	0	0.00	0	0.00	0	0.00
frontal	Parstriangularis	6	0.10	0	0.00	0	0.00	0	0.00	0	0.00	54	0.89
frontal	Parsorbitalis	0	0.00	0	0.00	0	0.00	4	0.12	0	0.00	642	18.59
frontal	Superiorfrontal	0	0.00	0	0.00	0	0.00	0	0.00	0	0.00	74	0.20
frontal	Rostralmiddlefrontal	14	0.06	0	0.00	0	0.00	8	0.03	0	0.00	3053	12.42
frontal	Medialorbitofrontal	0	0.00	0	0.00	0	0.00	5	0.06	27	0.30	0	0.00
frontal	Lateralorbitofrontal	0	0.00	0	0.00	0	0.00	51	0.39	5	0.04	665	5.05

Supplementary table 2: Brain atlas parcellations as defined by Desikan and colleagues (2007) and their statistical parameters, as uncovered by our GLMs on hand imitation scores on the different subsamples. Bayesian results indicating sufficient evidence/significant frequentist results are presented in bold.

	All hand			shared hand			exclusive hand		
**Label**	**BF**	**logBF**	**t-scores**	**BF**	**logBF**	**t-scores**	**BF**	**logBF**	**t-scores**
Thalamus-Proper	0.28	-1.27	-0.78	0.28	-1.29	-0.53	0.25	-1.40	-0.14
Caudate	0.30	-1.22	-0.85	0.28	-1.28	-0.56	0.26	-1.36	-0.34
Putamen	0.31	-1.18	-0.90	0.28	-1.28	-0.55	0.27	-1.32	-0.58
Pallidum	0.28	-1.26	-0.79	0.27	-1.31	-0.47	0.26	-1.36	-0.39
Hippocampus	0.31	-1.18	0.90	0.85	-0.17	1.71	0.25	-1.40	0.19
Amygdala	0.22	-1.53	-0.10	0.26	-1.34	0.41	0.24	-1.41	-0.15
Accumbens-area	0.22	-1.52	0.20	0.27	-1.29	0.52	0.30	-1.19	-0.70
Banks STS	0.37	-0.99	1.12	0.57	-0.55	1.42	0.43	-0.83	1.23
Caudalanteriorcingulate	0.22	-1.53	-0.16	0.25	-1.40	0.13	0.46	-0.77	-1.20
Caudalmiddlefrontal	0.75	-0.29	-1.69	0.99	-0.01	-1.81	0.28	-1.28	-0.49
Cuneus	**286.12**	**5.66**	4.14	1.63	0.49	2.12	**5000420.36**	**15.43**	**6.95**
Entorhinal	0.21	-1.54	-0.02	0.33	-1.10	0.85	0.24	-1.41	-0.01
Fusiform	0.22	-1.53	0.13	0.32	-1.14	0.78	0.26	-1.35	0.40
Inferiorparietal	6.04	1.80	2.78	3.01	1.10	2.45	**53.65**	**3.98**	3.71
Inferiortemporal	0.21	-1.54	0.01	0.31	-1.17	0.74	0.24	-1.41	0.05
Isthmuscingulate	0.44	-0.81	1.28	0.00	-	0.00	0.89	-0.12	1.77
Lateraloccipital	0.25	-1.41	0.55	0.28	-1.26	0.59	0.58	-0.55	1.46
Lateralorbitofrontal	0.25	-1.39	-0.58	0.25	-1.39	-0.18	0.37	-0.98	-1.02
Lingual	7.39	2.00	2.86	7.37	2.00	2.86	2.81	1.03	2.45
Medialorbitofrontal	0.24	-1.43	0.50	0.32	-1.14	0.79	0.33	-1.10	-0.92
Middletemporal	0.22	-1.52	-0.20	0.27	-1.32	0.45	0.25	-1.38	-0.21
Parahippocampal	0.22	-1.49	0.32	0.65	-0.43	1.52	0.27	-1.32	0.48
Paracentral	1.62	0.48	2.15	0.40	-0.92	1.07	**29.04**	**3.37**	3.44
Parsopercularis	15.15	2.72	-3.15	5.10	1.63	-2.70	2.65	0.98	-2.49
Parsorbitalis	0.47	-0.76	-1.33	0.50	-0.68	-1.30	0.30	-1.21	-0.66
Parstriangularis	2.02	0.70	-2.27	0.88	-0.12	-1.74	0.85	-0.16	-1.75
Pericalcarine	**23.90**	**3.17**	3.32	1.38	0.32	2.02	**2941.26**	**7.99**	4.96
Postcentral	0.23	-1.49	-0.34	0.40	-0.92	-1.07	0.63	-0.46	1.45
Posteriorcingulate	0.33	-1.12	0.98	0.36	-1.01	0.97	0.36	-1.01	1.00
Precentral	0.62	-0.47	-1.56	1.49	0.40	-2.07	0.25	-1.40	-0.21
Precuneus	**326.45**	**5.79**	4.18	2.90	1.06	2.43	**4822486.78**	**15.39**	**6.96**
Rostralanteriorcingulate	0.21	-1.54	-0.06	0.25	-1.39	0.20	0.38	-0.97	-0.99
Rostralmiddlefrontal	1.15	0.14	-1.96	0.53	-0.64	-1.35	0.85	-0.16	-1.65
Superiorfrontal	0.25	-1.38	-0.60	0.26	-1.36	-0.35	0.37	-1.00	-0.95
Superiorparietal	**143.13**	**4.96**	3.93	9.21	2.22	2.96	**191643.60**	**12.16**	**6.15**
Superiortemporal	0.22	-1.51	-0.27	0.25	-1.40	0.17	0.24	-1.41	-0.08
Supramarginal	0.44	-0.82	1.28	0.29	-1.24	0.62	14.71	2.69	3.18
Temporalpole	0.23	-1.49	0.34	0.34	-1.06	0.90	0.25	-1.40	-0.17
Transversetemporal	0.22	-1.52	-0.18	0.28	-1.26	-0.58	0.44	-0.83	1.03
Insula	0.57	-0.57	-1.48	0.43	-0.85	-1.14	0.39	-0.95	-1.19

Supplementary table 3: Brain atlas parcellations as defined by Desikan and colleagues (2007) and their statistical parameters, as uncovered by our GLMs on finger imitation scores on the different subsamples. Bayesian results indicating sufficient evidence/significant frequentist results are presented in bold.

	**All finger**			**shared finger**			**exclusive finger**		
**Label**	**BF**	**logBF**	**t-scores**	**BF**	**logBF**	**t-scores**	**BF**	**logBF**	**t-scores**
Thalamus-Proper	0.22	-1.51	-0.26	0.23	-1.48	-0.23	0.25	-1.39	0.27
Caudate	0.30	-1.19	0.88	0.24	-1.43	0.40	1.16	0.15	1.99
Putamen	2.41	0.88	2.35	0.91	-0.10	1.80	5.53	1.71	2.82
Pallidum	0.39	-0.94	1.16	0.33	-1.10	0.95	0.61	-0.49	1.53
Hippocampus	1.49	0.40	2.10	2.33	0.84	2.33	0.26	-1.35	0.37
Amygdala	0.94	-0.07	1.83	1.06	0.05	1.89	0.31	-1.18	0.79
Accumbens-area	0.74	-0.31	1.67	0.61	-0.49	1.52	0.36	-1.02	0.98
Banks STS	0.22	-1.49	-0.32	0.22	-1.50	-0.10	0.28	-1.29	-0.71
Caudalanteriorcingulate	1.49	0.40	2.10	0.51	-0.67	1.38	5.59	1.72	2.77
Caudalmiddlefrontal	0.21	-1.54	0.03	0.27	-1.31	-0.66	0.94	-0.07	1.85
Cuneus	0.96	-0.04	1.85	1.45	0.37	2.08	0.27	-1.31	0.47
Entorhinal	0.30	-1.21	0.86	0.33	-1.10	0.96	0.25	-1.37	0.35
Fusiform	0.33	-1.11	0.98	0.42	-0.87	1.20	0.24	-1.41	0.13
Inferiorparietal	0.25	-1.37	0.62	0.39	-0.94	1.14	0.57	-0.57	-1.40
Inferiortemporal	0.27	-1.30	0.73	0.35	-1.04	1.02	0.25	-1.38	-0.32
Isthmuscingulate	0.25	-1.41	-0.55	0.24	-1.43	-0.41	0.25	-1.40	-0.72
Lateraloccipital	0.46	-0.78	1.31	0.78	-0.24	1.70	0.47	-0.75	-1.20
Lateralorbitofrontal	2.54	0.93	2.38	1.25	0.22	1.99	2.48	0.91	2.42
Lingual	7.95	2.07	2.89	17.77	2.88	3.21	0.26	-1.35	-0.45
Medialorbitofrontal	1.79	0.58	2.20	1.53	0.43	2.11	0.25	-1.37	0.36
Middletemporal	0.22	-1.50	0.30	0.26	-1.34	0.60	0.28	-1.29	-0.54
Parahippocampal	0.22	-1.53	0.13	0.23	-1.48	0.23	0.25	-1.39	0.26
Paracentral	0.29	-1.24	0.82	0.34	-1.08	0.98	0.24	-1.41	0.10
Parsopercularis	0.24	-1.42	-0.51	0.37	-1.00	-1.07	0.41	-0.90	1.20
Parsorbitalis	0.75	-0.29	1.69	0.35	-1.05	1.01	5.24	1.66	2.77
Parstriangularis	0.28	-1.29	0.75	0.22	-1.50	0.05	2.11	0.75	2.35
Pericalcarine	0.80	-0.22	1.73	1.26	0.23	2.00	0.25	-1.40	0.24
Postcentral	0.26	-1.33	-0.69	0.27	-1.29	-0.68	0.28	-1.28	0.59
Posteriorcingulate	0.68	-0.39	1.62	0.91	-0.09	1.80	0.25	-1.38	0.31
Precentral	0.23	-1.48	-0.37	0.30	-1.21	-0.82	0.54	-0.61	1.47
Precuneus	0.39	-0.95	1.15	0.54	-0.62	1.42	0.26	-1.35	-0.20
Rostralanteriorcingulate	0.98	-0.02	1.86	0.39	-0.94	1.13	1.19	0.17	1.96
Rostralmiddlefrontal	0.49	-0.72	1.36	0.22	-1.50	0.03	**183.65**	**5.21**	4.14
Superiorfrontal	0.74	-0.30	1.68	0.27	-1.32	0.63	6.94	1.94	2.87
Superiorparietal	0.27	-1.31	0.72	0.41	-0.89	1.18	0.28	-1.27	-0.79
Superiortemporal	0.24	-1.42	0.52	0.27	-1.31	0.65	0.24	-1.41	0.13
Supramarginal	0.27	-1.32	-0.71	0.26	-1.35	-0.59	0.26	-1.35	-0.50
Temporalpole	0.90	-0.11	1.80	1.30	0.26	2.02	0.25	-1.40	0.22
Transversetemporal	0.22	-1.50	-0.29	0.23	-1.46	-0.32	0.26	-1.36	0.42
Insula	0.25	-1.37	0.62	0.23	-1.45	0.33	0.50	-0.70	1.41

Supplementary table 4: Detailed account of fibers connecting the finger area (rMFG) and hand areas according to a normative tractogram (HCP-842, [Yeh et al., 2018]). Total connections refers to fibers connecting the respective region pair regardless of the lesion cluster. Affected connections (abs.) documents the absolute number of disconnections between the region pair caused by the lesion cluster Affected connections (%) indicates the relative disconnection caused by the lesion cluster.

Pairwise connection		Total connections	Affected connections (abs.)	Affected connections (%)
rMFG	precuneus	368, (IFOF), 8 (cing.)	17 (IFOF), 1 (cing.)	4.6%, 12.5%
rMFG	cuneus	41 (IFOF)	0	0%
rMFG	pericalcarine cortex	612 (IFOF)	195 (IFOF)	31.3%
rMFG	superior parietal lobe	192 (IFOF), 45 (cing.), 6 (SLF)	69 (IFOF)	35.9%, 0%, 0%
rMFG	inferior parietal lobe	44 (IFOF), 15 (SLF)	40 (IFOF)	90%, 0%
rMFG	paracentral lobule	0	0	-

References

Desikan, R. S., Ségonne, F., Fischl, B., Quinn, B. T., Dickerson, B. C., Blacker, D., Buckner, R. L., Dale, A. M., Maguire, R. P., Hyman, B. T., Albert, M. S., & Killiany, R. J. (2006). An automated labeling system for subdividing the human cerebral cortex on MRI scans into gyral based regions of interest. *NeuroImage*, *31*(3), 968–980. https://doi.org/10.1016/j.neuroimage.2006.01.021

Yeh, F.-C., Panesar, S., Fernandes, D., Meola, A., Masanori;, Y., Fernandez-Miranda, J. C., Vettel, J. M., & Verstynen, T. (2018). Population-Averaged Atlas of the Macroscale Human Structural Connectome and Its Network Topology. *NeuroImage*, *178*, 57–68. https://doi.org/doi:10.1016/j.neuroimage.2018.05.027.
